# Modes of Action of Microbial Biocontrol in the Phyllosphere

**DOI:** 10.3389/fmicb.2020.01619

**Published:** 2020-07-14

**Authors:** Marie Legein, Wenke Smets, Dieter Vandenheuvel, Tom Eilers, Babette Muyshondt, Els Prinsen, Roeland Samson, Sarah Lebeer

**Affiliations:** ^1^Environmental Ecology and Applied Microbiology, Department of Bioscience Engineering, University of Antwerp, Antwerp, Belgium; ^2^Department of Plant and Microbial Biology, University of California, Berkeley, Berkeley, CA, United States; ^3^Laboratory for Integrated Molecular Plant Physiology Research, Department of Biology, University of Antwerp, Antwerp, Belgium

**Keywords:** biocontrol, phyllosphere, plant immune system, induced systemic resistance, antipathogenic mechanisms, plant pathogens, beneficial microbes, probiotics

## Abstract

A fast-growing field of research focuses on microbial biocontrol in the phyllosphere. Phyllosphere microorganisms possess a wide range of adaptation and biocontrol factors, which allow them to adapt to the phyllosphere environment and inhibit the growth of microbial pathogens, thus sustaining plant health. These biocontrol factors can be categorized in direct, microbe–microbe, and indirect, host–microbe, interactions. This review gives an overview of the modes of action of microbial adaptation and biocontrol in the phyllosphere, the genetic basis of the mechanisms, and examples of experiments that can detect these mechanisms in laboratory and field experiments. Detailed insights in such mechanisms are key for the rational design of novel microbial biocontrol strategies and increase crop protection and production. Such novel biocontrol strategies are much needed, as ensuring sufficient and consistent food production for a growing world population, while protecting our environment, is one of the biggest challenges of our time.

## Introduction

Pathogens and pests cause between 20% and 30% of global crop yield losses (Savary et al., 2019). To ensure a sufficient and consistent yield we depend on chemical crop protection and growth-promoting products such as pesticides, herbicides, and fertilizers. Many of these chemical products pose a threat to human health and the environment, which fuels a demand for safer products (Nishimoto, 2019). A promising alternative is the use of microbial based products that can protect crops against diseases. Such microbial products are classified under biological control agents, defined as “a natural enemy, antagonist, or other organism, used for pest control” (ISPM 05, International Standards for Phytosanitary Measures). Although biocontrol is a broad term, including eukaryotic biocontrol agents such as yeasts, fungi, beneficial insects, and other non-microbial pests, in this review we focus on bacterial biocontrol agents. We will use the term biocontrol agent defined similarly as probiotics, “live microorganisms which when administered in adequate amounts confer a health benefit on the host” (Hill et al., 2014). We use this interpretation of a biocontrol agent because it does not only focus on antagonizing the pathogen, but also on improving plant health. Moreover, this definition allows to draw parallels between probiotic and biocontrol research. We will focus on the mechanisms of bacterial biocontrol agents targeting microbial pathogens.

The phyllosphere, the above-ground surface of plants, is a complex ecosystem where microorganisms and the host plant interact extensively to create specific, yet dynamic, communities. Microbial communities inhabit both the external surfaces (epiphytes) as the internal spaces (endophytes) and these communities play an important role in protecting the plant against diseases. Pathogens often have an epiphytic phase before entering the plant cell or the apoplast (intercellular space) (Pfeilmeier et al., 2016). In this review, we focus on external leaf applications of biocontrol agents, unless otherwise specified.

**FIGURE 1:**
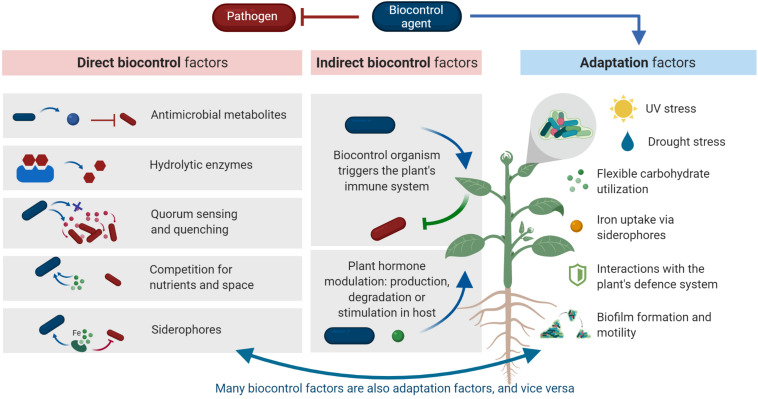
Overview of mechanisms described in this review. A successful biocontrol agent possesses biocontrol factors as well as adaptation factors. Biocontrol factors can be divided in direct and indirect interactions. Direct interactions are interactions directly between biocontrol agent (blue rod) and pathogen (red rod). Indirect interactions are interactions between biocontrol agent and host plant that lead to an enhanced immunity of the host plant against pathogen infection. Adaptation factors are factors that are required to adapt to the specific phyllosphere conditions, such as high levels of UV stress, low availability of water and nutrients and responses from the host immune system. Finally, adaptation factors are often biocontrol factors and vice versa, this is further explained in the text. Created with BioRender.com.

In analogy to a successful probiotic micro-organism, a successful biocontrol agent needs both specific adaptations that allow survival in the phyllosphere habitat (adaptation factors), as well as factors that contribute to the health of the host plant, by inhibiting the pathogen (probiotic or biocontrol factors) (Lebeer et al., 2008). To exert their beneficial properties, biocontrol agents need to be adapted to abiotic environmental factors as well as biotic host-specific factors. A general overview of environmental adaptation factors for the phyllosphere can be found in a review by Vorholt (2012). Adaptation factors are often overlooked in biocontrol research. However, low efficacy of biocontrol agents in field studies is often due to a lack of adaptation rather than a lack of biocontrol factors (Zeriouh et al., 2014; Salvatierra-Martinez et al., 2018). Moreover, a successful biocontrol agent needs a variety of adaptation and biocontrol factors to inhibit a pathogen and improve plant health (Köhl et al., 2019). Biocontrol factors can be related to direct or indirect microbial interactions (Figure 1). Direct interactions occur between the pathogen and the biocontrol agent. Indirect interactions are the interactions between the biocontrol agent and the host plant which improvesthe plant’s fitness, like its resistance to the disease. In this review, we will give an overview of direct and indirect biocontrol and adaptation mechanisms relevant for biocontrol in the phyllosphere. Furthermore, we will describe these mechanisms and the genetic basis in detail, and indicate whether these mechanisms have been validated in the field, *in vitro* or in greenhouse experiments. An overview of biocontrol and adaptation factors discussed in the text is given in Table 1.

**TABLE 1:** Overview of several known mechanisms by which phyllosphere microbes can inhibit pathogen growth.

*In vitro* screening	*In silico* screening	Compound	Mechanism/specific activity	Identified in	References	BC	A
**1.1 Antibiotic metabolites**							
Binary inhibitory interactions, purification and identification of compounds in supernatant	Screening for biosynthetic gene clusters using the antiSMASH tool	Antimicrobial secondary metabolites	Various	Various species	Helfrich et al., 2018	X	
	*srf*AC, *srf*AD	Lipopeptide, surfactin	Triggers biofilm formation	*Bacillus* spp.	Chen et al., 2007; Ongena and Jacques, 2008; Dunlap et al., 2013; Kim et al., 2015		X
	*fen*F, *myc*ABC	Lipopeptide, iturin	Interferes with lipid layers	*Bacillus* spp.	Chen et al., 2007; Ongena and Jacques, 2008; Dunlap et al., 2013; Kim et al., 2015	X	
	*pps*ABCDE	Lipopeptide, fengycin	Interferes with lipid layers	*Bacillus* spp.	Chen et al., 2007; Ongena and Jacques, 2008; Dunlap et al., 2013; Kim et al., 2015	X	
	*phz* gene cluster, *ehp* gene cluster	Phenazine	Interferes with histone acetylation and biofilm formation	*Pseudomonas* spp., *Pantoea* spp.	Giddens et al., 2002; Chin-A-Woeng et al., 2003	X	X
	*dda*A-I	Herbicolin I		*Pantoea vagans C9-1*	Kamber et al., 2012	X	
**1.2 Hydrolytic enzymes**							
Zymogram, or specific colorimetric assays	*chi*A, *chi*B, *chi*C or other genes encoding for glycosyl hydrolases from family 18 or 19 in the CAZy database	Hydrolytic enzymes: e.g., chitinase	Hydrolyses fungal cell wall	*Bacillus subtilis*	Essghaier et al., 2012	X	
	*msp1 (p75)*	Bifunctional peptidoglycan hydrolase/chitinase	Inhibits hyphae formation	*Lactobacillus casei* group species	Allonsius et al., 2019	X	
**1.3 Quorum quenching and sensing**							
	*nis* gene cluster (nisin), *spa* gene cluster (subtilin), *lux*I and *lux*R (AHLs)	Signalling molecules. Some gr- bacteria use bacteriocins (nisin and subtilin) that also have a signalling function	Quorum sensing	Nisin in *lactococcus lactis* and subtilin in *Bacillus subtilis*	Kleerebezem, 2004		X
Screening of bacteria interfering with the transcription of a reporter gene, induced by the signalling molecule of interest	*carAB* (degradation signaling molecule of *Xylella fastidiosa*), *aii*A (AHL lactonase)	Enzymes involved in degradation signalling factors	Quorum quenching	*Bacillus, Paenibacillus, Microbacterium, Staphylococcus*, and *Pseudomonas*	Newman et al., 2008; Morohoshi et al., 2009; Alymanesh et al., 2016	X	
**1.4 Competition for nutrients and space**							
Carbon source profiling and calculation of NOI	Genes related in carbohydrate metabolism (e.g., glycosyl hydrolases), or transport (e.g., Tonb receptors), using the CAZy database	Enzymes ensuring flexible carbohydrate metabolism, e.g., high diversity of TonB receptors	Increased competitiveness in a carbon limited environment	*Sphingomonas* spp.	Delmotte et al., 2009	X	X
Selective media with methanol as sole carbon source	*mxa*F	Conserved enzyme responsible for methanol dehydrogenase	Methylotrophy, increased adaptability in a carbon limited environment	*Methylobacterium* spp.	Mcdonald and Murrell, 1997		X
**1.5 Siderophores**							
Plate assay with indicator for detection of siderophores (Chrome azurol S assay)	Screening for siderophore gene clusters, using antiSMASH	Siderophores	Primary function is iron chelation. Siderophores can also have antibacterial properties trough the production of ROS and play a role in motility on the phyllosphere	*Pseudomonas protegens* CS1	Burbank et al., 2015; Santos Kron et al., 2020	X	X
**2.1 Modulation plant hormone levels**							
Colorimetric assays	*iac* gene cluster	Enzymes responsible for the degradation of indole-3-acetic acid (IAA)	IAA is used as an energy source and modulation of IAA levels induces physiological changes in the plant	*Pseudomonas putida* 1290	Leveau and Gerards, 2008	X?	X
HPLC analysis of extracts of the supernatant	*ipd*C*/ald*H *or dcc/ald*H *or iaa*M*/iaa*H *or nth*A	Enzymes involved in the production of IAA, several pathways possible, described in text	Modulation of IAA levels can enhance plant growth, enhanced protection against pathogens has not been demonstrated so far	*P. agglomerans*	Brandl et al., 2001; Duca et al., 2014; de Souza et al., 2019	X?	X
Cultivation with 1-aminocyclopropane-1 carboxylate as nitrogen source and by measuring production α-ketobutyrate (end-product) spectrophotometrically	*acd*S or *acc*D	Enzymes responsible for lowering ethylene levels	1-aminocyclopropane-1-carboxylate deaminase, modulation of ethylene levels induces physiological changes in the plant. Enhanced protection against pathogens has not been demonstrated so far	*Methylobacterium* spp., *R. fascians*	Chinnadurai et al., 2009; Francis et al., 2016	X?	X
HPLC analysis of extracts of the supernatant	*fas*4 or *IPT*	Enzymes responsible for production cytokinins	Isopentenyl transferase, modulation of cytokinins levels induces physiological changes in the plant. Enhanced protection against pathogens has not been demonstrated so far	*Methylobacterium* spp., *R. fascians*	Madhaiyan et al., 2006; Francis et al., 2016; Jorge et al., 2019	X?	X
**2.2 Induced systemic response**							
Transcriptomics of the host plant	Creation of a MAMP database, compare between beneficial and pathogenic microbes	MAMPs that trigger an immune response, that increases protection against pathogens	Detection results in immune response	*Sphingomonas melonis* fr1	Ryffel et al., 2016; Vogel et al., 2016	X	X
Transcriptomics of the host plant	Creation of an effector database, screening for type III secretion system gene clusters	effectors that trigger an immune response, that increases protection against pathogens	Detection results in immune response	*Pseudomonas* spp., *Parabulkholderia* sp.	Stringlis et al., 2019; Herpell et al., 2020	X	X

*The table includes (i) information on *in vitro* assays to test for the presence of these mechanisms, (ii) known genes involved in these mechanisms (*in silico* screening), (iii) the compound and (iv) the mechanism resulting in antipathogenic activity, (v) microbes in which the mechanism has been identified, (vi) references and the last two columns indicate whether the mechanism is (vii) a biocontrol factor (BC) and/or (viii) an adaptation factor (A). The screening methods, strains and references are not exhaustive but rather examples, which are also discussed in the text. The table follows the same structure as the review.*

## The Phyllosphere Microbial Habitat

The phyllosphere is inhabited by a complex and dynamic community. The composition of this community depends on which microbes reach the phyllosphere in addition to abiotic factors such as climate, season and surrounding land use, and biotic factors such as leaf characteristics and host plant species (Maignien et al., 2014; Agler et al., 2016; Laforest-Lapointe et al., 2016; Smets et al., 2016). Microbes arrive on the phyllosphere rather stochastically via the air, soil, rain or insects. However, only selected taxa successfully colonize the phyllosphere (Maignien et al., 2014). Frequently occurring genera in phyllosphere communities are *Methylobacterium*, *Sphingomonas*, and *Pseudomonas* (Delmotte et al., 2009; Vorholt, 2012). These common phyllosphere bacteria possess specific adaptation factors to the phyllosphere. For example, *Methylobacterium* spp. have adapted to the low-nutrient environment by metabolizing single-carbon compounds such as methanol (Kutschera, 2007). *Sphingomonas* spp. cope with the scarcity of nutrients by being able to metabolize a wide range of carbon sources (Delmotte et al., 2009). *Pseudomonas* spp. use flagellar motility to reach more favorable sites (Haefele and Lindow, 1987), synthesize the biosurfactant syringafactin to increase the water availability on leaf surfaces (Hernandez and Lindow, 2019), and use effectors to leak water from the cells into the apoplast (Xin et al., 2016).

## Direct Interactions

### Antibiotic Metabolites and Binary Inhibitory Interactions

A key first step in the identification of novel biocontrol agents is the screening of antagonistic activities. Such screenings are increasingly applied at a larger scale. For example, Helfrich et al. (2018) recently screened more than 200 leaf isolates from *Arabidopsis thaliana* for binary inhibitory interactions, novel antagonistic strains and interesting metabolites. Most of these strains (88%) engaged in such inhibitory interactions. The orders *Bacillales* and *Pseudomonadales* were especially strong inhibitors, making up only 8% of the tested isolates but engaging in over 60% of the observed inhibitions. Most of the inhibitions also took place between distinct phylogenetic groups rather than within the same family or genus. Genome analysis using the antiSMASH tool (Blin et al., 2019) revealed that many of the inhibitory strains contained more biosynthetic gene clusters than average. These clusters can encode for metabolites with inhibitory effects. The top inhibitor of the collection, *Brevibacillus* sp. Leaf182, was shown to produce several non-ribosomal peptides with antimicrobial activity, such as marthiapeptide A (an anti-infective and cytotoxic polythiazole cyclopeptide previously isolated from deep-sea *Marinactinospora thermotolerans*), streptocidin D (a cyclic decapeptide antibiotic from the tyrocidine family, named after tyrothricin, the first commercially available antibiotic containing tyrocidine and gramicidin), and an unusual lysophospholipid (a bioactive molecule that possesses a large polar or charged head and a single hydrophobic carbon chain), which was active against Gram-negative bacteria. Previously, biocontrol activity by a *Brevibacillus brevis* strain against *Botrytis cinerea* had been observed in the phyllosphere of Chinese cabbage (Edwards and Seddon, 2001). This strain produces the antibiotic gramicidin S, another cyclic antibiotic non-ribosomal decapeptide and major constituent of tyrothricin. Comparison of biocontrol activity with an antibiotic-negative mutant and pure gramicidin S showed that gramicidin S was the mechanism behind the observed biocontrol.

The *Pseudomonas* genus is frequently found in the phyllosphere in relatively high abundances (Delmotte et al., 2009; Maignien et al., 2014). The *Pseudomonas* genus includes several commercialized biocontrol strains, such as *Pseudomonas chlororaphis* MA342 and *Pseudomonas* sp. DSMZ 13134. However, also several plant pathogens belong to this genus, such as the model phyllosphere pathogen *Pseudomonas syringae* pv. *tomato* DC3000 (Innerebner et al., 2011). Biocontrol *Pseudomonas* strains have been observed to directly inhibit the growth of a wide variety of pathogens (such as *P. syringae* and *B. cinerea*) in lab and in field experiments (Völksch and May, 2001; Romero et al., 2016; Simionato et al., 2017). Biocontrol activity of *Pseudomonas* spp. is often attributed to the production of phenazines, a group of heterocyclic nitrogen-containing secondary metabolites (Chin-A-Woeng et al., 2003). Biosynthesis in *Pseudomonas* spp. is encoded by the *phz* gene cluster. Phenazines inhibit the growth of a variety of fungal pathogens, such as *B. cinerea* and *Fusarium oxysporum* [a more detailed overview is given in Chin-A-Woeng et al. (2003)]. The antifungal mode of action of phenazines is multifaceted. For example, Chen et al. (2018) demonstrated that phenazines inhibit mycelial growth of *Fusarium graminearum* by interference with fungal histone acetylation, and are involved in the formation of a bacterial biofilm on the hyphae, further decreasing pathogenicity. Biofilm formation on fungal hyphae is a widespread trait in soil bacteria (Guennoc et al., 2018). More studies are needed to determine how frequent this occurs in the phyllosphere. Next to phenazines, many other potential biocontrol metabolites have been identified in *Pseudomonas* spp. such as siderophores (see section “Siderophores”), 4-hydroxy-2-alkylquinolines (Yasmin et al., 2017), volatile compounds such as cyanide and other volatile organic compounds (Bailly and Weisskopf, 2017), and cyclic lipopeptides (non-ribosomal peptides) and rhamnolipids (glycolipids synthesized in a three-step biosynthetic pathway including *rhl*ABC enzymes) (Nielsen et al., 2006; Strano et al., 2017; Yasmin et al., 2017). Rhamnolipids are effective against zoosporic root-infecting pathogens such as *Phytium* and *Phytotophtera* spp. Furthermore, spraying purified rhamnolipids on leaves of Arabidopsis triggers an immune response in the host characterized by the accumulation of signaling molecules and defense genes (Sanchez et al., 2012) (this is an indirect biocontrol mechanism and is further discussed in the section “Plant Hormones”). Interestingly, cyclic lipopeptides and rhamnolipids are also biosurfactants. Biosurfactants generally improve surface motility, biofilm formation and colonization of plant surfaces. Therefore, these adaptation factors could play an important role in the effectiveness of *Pseudomonas* biocontrol agents. Although, to our knowledge, the importance of rhamnolipids in adaptation, has not yet been investigated in the phyllosphere. Recently, Santos Kron et al. (2020) investigated the role of three antibiotic compounds in the antagonism by *Pseudomonas orientalis* F9 via experiments with mutants deficient in the production of the siderophore pyoverdine (also see section “Siderophores”), safracin (a tetrahydroisoquinoline alkaloid) and phenazine. *In vitro* double-layer assays showed antagonism against *Erwinia amylovora* and three *P. syringae* pathovars by the parental strain *P. orientalis* F9 and surprisingly also by the pyoverdine and phenazine deficient mutants. Only the mutant deficient in safracin production did not inhibit the pathogens. This indicates that safracin rather than pyoverdine and phenazine was causing the *in vitro* antagonism. In contrast, all mutants were able to inhibit the pathogen *Pythium ultimum, in vivo* in a soil microcosm and *E. amylovora, in vitro* in a detached blossom assay. These unexpected results indicate that the biocontrol mechanism of *P. orientalis* is versatile and that other mechanisms could play a role in the observed biocontrol. Recently, Bernal et al. (2017) described the use of a type VI secretion system for the secretion of the Rhs effector Tke2 in *Pseudomonas putida*. The secretion of this effector was shown to be responsible for inhibiting *P. syringae*, *Xanthomonas campestris*, *Pectobacterium carotovorum*, and *Agrobacterium tumefaciens in vitro*, as well as reducing colonization of *X. campestris* and reducing disease symptoms on *Nicotiana benthamiana* leaves. Furthermore, Chen et al. (2016) described the secretion of the antimicrobial siderophore pyoverdine by a type VI secretion system, which inhibits *Xanthomonas oryzae* pv. *oryzae* (see also section “Siderophores”). Many *Pseudomonas* spp., both pathogenic as non-pathogenic, as well as other Gram-negative phyllosphere bacteria, harbor type VI secretion systems, suggesting that these are an adaptation factor (Bernal et al., 2018).

Less frequent inhabitants of the phyllosphere, but often used in commercial biocontrol products, are *Bacillus* spp. (Ongena and Jacques, 2008). Bacilli isolated from the phyllosphere often engage in inhibitory interactions with other microbial competitors (Helfrich et al., 2018) and their ability to form resistant endospores facilitates their formulation and shelf life (Ongena and Jacques, 2008). *Bacillus subtilis* and *Bacillus amyloliquefaciens* are the two most described biocontrol agents in this genus thus far. *B. subtilis* strains inhibit a wide range of pathogens, both fungal and bacterial, such as *F. graminearum* (Wang et al., 2007), *B. cinerea* (On et al., 2015), *Alternaria* spp. (Ali et al., 2016), *X. campestris*, and *P. carotovorum* (Zeriouh et al., 2011). The antipathogenic activity of bacilli has mainly been attributed to the synthesis of non-ribosomal peptides and polyketides (Ongena and Jacques, 2008; Chen et al., 2009). The three classes of non-ribosomal lipopeptides, surfactin, iturin, and fengycin, often act in a synergistic manner. Interestingly, surfactins produced by *B. subtilis* do not appear to play a role in the antipathogenic activity *in vitro*, whereas they are necessary for biocontrol *in planta* (Zeriouh et al., 2014). Surfactins trigger biofilm formation, allowing *B. subtilis* to successfully colonize the phyllosphere in sufficient numbers and to manage the release of antimicrobial compounds. Therefore, surfactins are rather adaptation factors than biocontrol factors. Wei et al. (2016) confirmed that *B. subtilis* QST 713, which is used in commercial products, was able to colonize the leaf surface in sufficient numbers. However, despite successful colonization of the phyllosphere, difficulty to colonize new leaves (i.e., dispersal), limited the biocontrol potential of this product. Fengycins and iturins are mostly active against filamentous fungi, by interfering with the lipid layers and altering cell membrane structures (Ongena et al., 2007), but also against the Gram-negative pathogens *X. campestris* pv. *cucurbitae* and *P. carotovorum* subsp. *carotovorum* (Zeriouh et al., 2011). *B. amyloliquefaciens* strains have been proven to be successful biocontrol agents in the field for a wide range of pathogens, such as *Sclerotinia sclerotiorum* causing canola stem rot (Fernando et al., 2007), and fusarium head blight on durum wheat (Schisler et al., 2002). Salvatierra-Martinez et al. (2018) described biocontrol activity of two *B. amyloliquefaciens* strains against *B. cinerea* on tomato plants. These two trains had similar antagonistic effect *in vitro*, while strain BBC047 showed better results *in planta*. BBC047 was also able to produce a robust biofilm and maintain higher population density over time on the plants. Therefore, it is assumed that adaptation factors explain why strain BBC047 is a more effective biocontrol agent. The genomes of biocontrol *B. amyloliquefaciens* strains contain several gene clusters encoding for the three lipopeptides surfactin, iturin and fengycin, and polyketide compounds, such as bacillaene, macrolactin and difficidin (Chen et al., 2007; Dunlap et al., 2013; Kim et al., 2015). A clear overview of the secondary metabolite synthetase gene clusters in the genome of *B. amyloliquefaciens* AS 43.3 is given in Dunlap et al. (2013). Chen et al. (2009) demonstrated that in the mix of these antimicrobial metabolites, the polyketide difficidin and the dipeptide bacilysin, are most important for biocontrol against *E. amylovora* on apple blossoms. This was proven *in planta* by applying three mutants of the commercial strain FZB42 on detached apple blossoms, one mutant deficient in production of difficidin, the second unable to synthesize non-ribosomal lipopeptides and polyketides, and a third double mutant deficient in polyketide and bacilysin synthesis. Similarly, Wu et al. (2015) also show the role of difficidin and bacilysin from strain FZB42 in the antagonistic mechanism against *X. oryzae* pv. *oryzae* and *X. oryzae* pv. *oryzicola.* Moreover, microscopic techniques revealed that difficidin and bacilysin cause changes in the cell wall of *Xanthomonas* spp.

The genus *Pantoea* contains several plant pathogens, as well as biocontrol agents effective against a range of pathogens such as *B. cinerea*, *X. campestris*, and, the most extensively studied, *E. amylovora* [as reviewed by Walterson and Stavrinides (2015)]. Several antibiotics, such as pantocins (Smits et al., 2019), herbicolins (Kamber et al., 2012), and phenazines (Giddens et al., 2003), have been identified to play a role in the inhibition of *E. amylovora*. Stockwell et al. (2002) compared biological control of *E. amylovora* in field conditions by *Pantoea agglomerans* (syn. *Erwinia herbicola*) Eh252, known to produce only one antibiotic, and by its near-isogenic derivative, strain 10:12. Strain 10:12 is deficient in the production of mccEh252, involved in the synthesis of microcin C7. Strain Eh252 reduced the incidence of fire blight more effectively then 10:12. However, the mutant strain still protected the plants more effectively than a mock treatment, indicating that other mechanisms also contribute to biocontrol. The antibiotic herbicolin I was identified and characterized in *Pantoea vagans* C9-1 via the construction of a mutant library (Kamber et al., 2012). The herbicolin I biosynthetic gene cluster responsible *dda*A-I is located on the plasmid pPag2. Remarkably, this cluster was not detected in many other biocontrol strains. Using a similar approach, another antibiotic gene cluster, PNP-1 was identified in *Pantoea ananatis* BFT175, also effective against *E. amylovora* (Walterson et al., 2014). The PNP-1 cluster shows similarities to a gene cluster encoding for phenazine in *Pseudomonas chloraphis*. Previously, the *ehp* gene cluster encoding for phenazine synthesis had been identified in the genome of *P. agglomerans* Eh1087 (Giddens et al., 2002). However, the PNP-1 gene cluster was not found in other *Pantoea* spp., indicating again the diversity of antibiotics in this genus.

Formulating antimicrobial metabolites into a plant protection product, without the living microorganism could result in a more convenient and cheaper product. Furthermore, the problem of limited biocontrol activity due to a low survival rate of the biocontrol agent would be solved. The formulation of a product with live bacteria is challenging, the drying process needs to be optimized to ensure a long shelf life and to minimize the loss of biocontrol activity (Broeckx et al., 2016). However, the use of live microorganisms does have advantages too. Firstly, the persistence of the metabolite in the environment. Antimicrobial metabolites can degrade rapidly in field conditions and would require frequent applications, while applying a living organism might need fewer. Secondly, antagonists are likely to acquire resistance toward a frequently applied metabolite. Live microorganisms and even consortia of live microorganisms have the advantage of producing various active molecules and thus reducing the chance of resistance. Finally, live microorganism can improve the health of plants not only via antimicrobial metabolites but via other direct and indirect mechanisms, as described in the next paragraphs.

### Hydrolytic Enzymes

Production of chitinases, as well as other cell wall degrading enzymes, such as β-1,3-glucanase, is a common defense mechanism of plants (Boller, 1993). Microbes can also produce chitinases, which are an important biocontrol mechanism in the rhizosphere (reviewed by Veliz et al., 2017). Their importance in the rhizosphere indicates the potential of further studying the microbial chitinase activity on the phyllosphere. It has been demonstrated that *B. subtilis* J9 strain protects strawberry plants against *B. cinerea* in field conditions and that this strain produces extracellular chitinase and protease (Essghaier et al., 2012). Recently, we observed that certain lactobacilli can inhibit hyphae formation of fungi *in vitro* by producing bifunctional enzymes with chitinase/peptidoglycan hydrolase activity (Allonsius et al., 2019). Lactobacilli are not typical phyllosphere inhabitants, and often have a low survival rate (Miller et al., 2019). However, they have been shown to dominate the endosphere of *Origanum vulgare* plants (Pontonio et al., 2018) and have been correlated negatively with disease symptoms of leaf spot on cucumber plants, presumably caused by *P. syringae* pv. *lachrymans* (Luo et al., 2019). Next to the production of hydrolytic enzymes by the biocontrol agents themselves, microbes can induce the production of chitinases in the host plant, a common defense reaction in plants. Inhibition of a pathogen by triggering a defense reaction in the host is further discussed in section “Indirect Interactions.”

### Quorum Sensing and Quenching

Quorum sensing systems are systems by which bacteria change their behavior once a certain concentration threshold of signaling molecules is passed. In the phyllosphere, signaling molecules mediate behavior that enables bacteria to survive on the leaf surface, such as biofilm development, adhesion, motility, and production of cell-wall-degrading enzymes. Pathogenic bacteria use quorum sensing to measure their population size and regulate the moment to enter the apoplast or plant cell (Pfeilmeier et al., 2016; Leach et al., 2017). Gram-negative bacteria often use N-acyl-homoserine lactones (AHLs) as signaling molecules, which are synthesized by AHL synthase (*luxI*) and detected by a transcriptional regulator (*luxR*). Interestingly, AHL molecules can also trigger a response in the host plant (Delalande et al., 2005; Sieper et al., 2014), which is further discussed in the section “Indirect Interactions.” Gram-positive bacteria do not make use of AHL systems, but typically use small post-translationally processed peptides as signal molecules or diffusible signal factors (see further in this section). A wide variety of small communication peptides exist, and these peptides sometimes have other functions as well. For example, *Lactococcus lactis* and *B. subtilis* produce the antibiotic lantipeptides nisin and subtilin, respectively, which are also involved in quorum sensing (Kleerebezem, 2004). Both *B. subtilis* (Wei et al., 2016) and *L. lactis* (Trias et al., 2008) can survive in the phyllosphere and even have biocontrol characteristics. However, involvement of the bifunctional peptides nisin and subtilin in the biocontrol activity on the phyllosphere has not been described. Therefore, it would be interesting to investigate their role in the biocontrol mechanism of these bacteria.

Interestingly, non-pathogenic bacteria use the same signaling molecules as pathogens and can thereby contribute to disease development or inhibition, depending on the way of interfering. A shared quorum sensing system using AHL-signal molecules was observed between the symbiotic bacteria *P. agglomerans*, *Erwinia toletana* and *Pseudomonas savastanoi* pv. *savastanoi*, the causative agent for knot disease in olive trees (Hosni et al., 2011). The symbionts, or in this case opportunistic pathogens, benefit from the niche created by disease development by the pathogen and thus participate in its communication system. By cooperating with the pathogen, *E. toletana* and *P. agglomerans* aggravated the infection in the olive trees in this study (Hosni et al., 2011). In contrast, other *P. agglomerans* strains showed biocontrol activity against the pathogen *P. syringae* pv. *tomato* in tomato plants (Morella et al., 2019), but it is at present not known whether quorum sensing could be involved. It remains to be determined whether actual biocontrol agents can have this effect on target and non-target pathogens.

Next to cross-communication by producing the same signaling molecules, bacteria can degrade each other’s signals, also known as quorum quenching. Strains belonging to the genera *Bacillus, Paenibacillus, Microbacterium, Staphylococcus*, and *Pseudomonas* are able to rapidly degrade the diffusible signal factor, cis-11-methyl-2-dodecenoic acid. This signal is involved in the regulation of virulence of *Xanthomonas* spp. and *Xylella fastidiosa* in a quorum-sensing AHL-independent way (Newman et al., 2008). In the quorum-quenching strains, the genes *car*AB, involved in synthesis of carbamoylphosphate, a precursor for pyrimidines and arginine, were identified to be required for the rapid degradation of this diffusible signal factor. Bacteria containing the *car*AB genes could reduce disease incidence and severity of *X. campestris* pv. *campestris* in a detached leaf assay with mustard, cabbage and turnip plants, and of *X. fastidiosa* when co-inoculated into the xylem of grape stems. Furthermore, Wu et al. (2015) showed that difficidin and bacilysin produced by *B. amyloliquefaciens* FZB42 (see section “Antimicrobial Metabolites”) can downregulate the expression of several virulence genes in *X. oryzae*, including *rpf*F, involved in the production of a diffusible signal factor.

Morohoshi et al. (2009) screened 109 isolates from the potato phyllosphere for the ability to degrade several short-chain and long-chain AHLs, as Gram-negative pathogens use AHLs as a signaling molecule to regulate their virulence. They screened the isolates *in vitro* by using AHL biosensors, i.e., bacteria that respond to the presence of AHLs by producing a reporter protein. One of the enzymes involved in AHL degradation is AHL-lactonase, encoded by the *aii*A gene, initially identified in *Bacillus* spp. *Microbacterium testaceum* strains StLB018 and StLB037 tested positive for AHL degradation and decreased disease symptoms in potato tissue caused by *P. carotovorum* subsp. *carotovorum*. In contrast, *M. testaceum* ATCC 15829, lacking AHL-degrading activity, did not decrease disease symptoms, indicating that quorum quenching was the mode of action of biocontrol. Alymanesh et al. (2016) used a similar method to screen isolates from the phyllosphere and rhizosphere from saffron, fig, and pomegranate, for the degradation of the AHL 3-oxo-C6-HSL. They concluded that quorum quenching is a common trait among the isolates tested and is most often observed in *Pseudomonas* spp. These *Pseudomonas* isolates with strong quorum quenching activity also showed biocontrol activity against *P. carotovorum* subsp. *carotovorum in vitro* and on potato tubers.

### Competition for Nutrients and Space

Phyllosphere bacterial community sizes are limited by low carbon availability on the leaf surface (Mercier and Lindow, 2000). Therefore, carbon competition will likely play an important role in the community structure. Microcosm experiments show that “invaders,” such as introduced biocontrol agents, with a similar metabolism as the resident species are strong competitors in environments with a low resource availability, whereas fast-growing species have an advantage when resource availability is high (Yang et al., 2017).

The dominant carbohydrates available on the leaf surface are sucrose, fructose and glucose. These sugars are specifically altered after epiphytic leaf colonization by *Sphingomonas melonis* or the pathogen *P. syringae* pv. *tomato*, but only to a minor extent by *Methylobacteria* (Ryffel et al., 2016). Phyllosphere bacteria have developed different strategies to utilize all possible carbon sources available. Methylotrophs, such as *Methylobacteria*, have specialized in the utilization of single carbon compounds, such as methane and methanol. Therefore, they do not rely as much on the available sugars on the phyllosphere (Kutschera, 2007). Methylobacteria even modulate the release of methanol, which is released as plant cells expand, by encouraging plant growth via the production of plant hormones (see further, section “Plant Hormones”) (Kutschera, 2007). The *mxa*F gene, which contains the active site of a methanol oxidation complex, was found to be highly conserved among methylotrophs and is an appropriate probe to screen for methylotrophy (Mcdonald and Murrell, 1997). Methylotrophy is thus an important adaptation factor for some phyllosphere bacteria. However, methylotrophs are not likely to inhibit pathogens by competing for nutrients. Nevertheless, Methylobacteria can possess other biocontrol mechanisms such as antimicrobial metabolites (Kwak et al., 2014) or indirect mechanisms by triggering plant immunity (see further in section “Plant Immunity”) (Madhaiyan et al., 2006).

Another adaptation strategy is the ability to scavenge for a wide variety of carbon sources. The presence of a high variety of TonB receptors in the phyllosphere proteome has been suggested as an indication that the residing species can metabolize a wide variety of carbon compounds (Delmotte et al., 2009). Indeed, TonB receptors are involved in the transport of carbohydrates, siderophores, and vitamin B_12_, in Gram-negative bacteria (Schauer and Kutschera, 2013). Blanvillain et al. (2007) noted that bacteria expressing a high variety of TonB receptors, but belonging to various taxonomical lineages, share the ability to metabolize a wide variety of carbohydrates. The overrepresentation of TonB receptors in *Xanthomonas* spp. appears to facilitate their survival in the phyllosphere by making them competitive nutrient scavengers (Blanvillain et al., 2007). Additionally, community proteogenomics of the phyllosphere of *Arabidopsis*, clover, and soybean assigned a high proportion and great variety of TonB receptors to *Sphingomonas* species. This high abundance of TonB receptors is thought to allow *Sphingomonas* spp. to be more successful than other Gram-negative bacteria to withstand the carbon-stressed environment and account for their success on the phyllosphere in terms of their relative abundance (Delmotte et al., 2009). Innerebner et al. (2011) tested 17 *Sphingomonas* strains on the phyllosphere of *A. thaliana* for their ability to suppress disease symptoms of the pathogen *P. syringae* pv. *tomato* DC3000. All seven phyllosphere isolates, and four out of five rhizosphere isolates, protected the plant against developing disease symptoms. On the other hand, four out of five *Sphingomonas* non-plant isolates (isolated from air, dust, or water), did not protect the host plant against *P. syringae* infection. Carbon-source profiling of two protective and two non-protective strains suggested that substrate competition plays a role in the observed antagonistic effect. It would be interesting to verify whether the difference in carbon-source utilization is a result of a higher TonB diversity and whether plant-associated *Sphingomonas* spp. typically have a higher TonB diversity in comparison to other *Sphingomonas* spp.

The niche-overlap index (NOI) is a measure that can be used to quantify the similarity in carbon source profile of two microbes (Wilson and Lindow, 1994). Wilson and Lindow (1994) calculated the NOI as the number of carbon sources that both strains utilize as a proportion of the total number of carbon sources utilized by one strain. They demonstrated that the NOI of the epiphytic bacteria *Pseudomonas fluorescens, P. agglomerans, Stenotrophomonas maltophilia*, and *Methylobacterium organophilum* correlated inversely with their ability to coexist with the pathogen *P. syringae* on the phyllosphere of beans (*Phaeseolus vulgaris*). In another study, the NOI of 36 non-pathogenic phyllosphere bacteria were correlated with the ability to suppress disease caused by *P. syringae* pv. *tomato* (Ji and Wilson, 2002). These studies confirm the hypothesis made by Lindow (1987) that “antagonism due to competition of one strain with another would increase proportionally to the overlap of their ecological niche.” This hypothesis was formulated based on a field study where ice nucleation-deficient *P. syringae* mutants successfully antagonized the *P. syringae* wild-type strain in field conditions when the mutant was applied to the plants two days before the wild-type strain (Lindow, 1987). Under such conditions, the mutants could successfully outcompete the wild-type strain and a reduction of the frost injury to the plants was noted. However, the mutants had the advantage of being able to occupy the ecological niche first. Priority effects do play an important role in competition between microbes and in the assembly of phyllosphere communities (Maignien et al., 2014). Therefore, some biocontrol agents are more effective as a preventive measure and less so as a treatment.

Berg and Koskella (2018) tested the antipathogenic properties of both a natural phyllosphere community and a simplified synthetic phyllosphere community (comprising of 12 bacterial strains), against *P. syringae* pv. *tomato*. Both the natural as the synthetic community protected the plant against the pathogen. The authors observed that addition of fertilizer to the soil canceled the observed pathogen protection of the synthetic community, but not of the natural community. Microbial loads on the leaves did not increase significantly due to fertilization. The authors hypothesize that fertilization resulted in an increase in phyllosphere nutrient availability. The synthetic communities were all cultured on KB medium before application on the plants. This is a medium on which *P. syringae* also grows well. This might have caused selection for metabolically similar strains, which would increase antagonism due to nutrient competition (cfr. Lindow, 1987). Nutrient competition might therefore play a more prominent role in the synthetic communities than in the more diverse natural communities, where other modes of action could possibly dominate. This hypothesis on nutrient-dependent effects provokes two novel research questions. Firstly, does soil fertilization increase nutrient availability in the phyllosphere and secondly, how does this have an impact on biocontrol in the phyllosphere in field conditions?

### Siderophores

Apart from carbon sources, iron is often a limiting element in phyllosphere microbial communities. Siderophores are secreted by microorganisms to bind and transport iron into the cell. Siderophore production is essential for the epiphytic fitness of *P. syringae* pv. *syringae* 22d/93, a strain with biocontrol activity against the pathogen *P. syringae* pv. *glycinea* (Wensing et al., 2010). Interestingly, when inoculated in wounded leaves, siderophore production by the commensal had no effect on its own population size nor on the population size of the pathogen. This indicates that iron was not a limiting element in wounded plant cells. Siderophore production is thus not a biocontrol mechanism of importance for *P. syringae* pv. *glycinea*, when the pathogen rapidly penetrates living tissue. However, siderophore production is an important adaptation factor for biocontrol agent *P. syringae* pv. *syringae* 22d/93, as 10 days post inoculation, the population size of a siderophore-negative mutant was 2 orders of magnitude lower than that of the wild-type. Furthermore, a role for siderophores in the induced systemic resistance (ISR) (see section “Induced Systemic Responses”) has been reported in several systems (Bakker et al., 2007). It is not excluded that the wounding in the experiment by Wensing et al. (2010) triggered ISR, via host jasmonic acid (JA) and ethylene mediated pathways (see section “Plant Hormones”). The wounding switched off the necessity for an additional siderophore triggered ISR and the strain did not exert any biocontrol activity in the wounded plants.

Siderophores can have alternative functions in addition to iron scavenging, such as non-iron metal transport, sequestration of toxic metals, signaling, protection from oxidative stress, and antibiotic activity. The latter occurs by attaching a bactericidal ‘warhead’ on a siderophore which is then taken up by the antagonized bacterium (Kramer et al., 2019). The siderophore enantio-pyochelin, produced by *Pseudomonas protegens* CS1, isolated from the lemon tree phyllosphere, showed antagonistic activity *in vitro* and in the phyllosphere of lemon plants against the pathogen *Xanthomonas citri* subsp. *citri* (Michavila et al., 2017). Additions of iron and ascorbic acid indicated that not competition for iron but oxidative stress, induced by the formation of reactive oxygen species (ROS) from pyochelin, was the mechanism of action for the observed antimicrobial activity. Indeed, ascorbic acid was able to counteract the antimicrobial activity of ROS while addition of iron had almost no effect. In contrast, experiments with *P. orientalis* F9 and a mutant deficient in the production of siderophore pyoverdine (also see section “Antibiotic Metabolites”) showed that the mutant was still able to antagonize *E. amylovora* and three *P. syringae* pathovars *in vitro*, as well as *E. amylovora* on a detached flower assay and *P. ultimum* in a soil microcosm assay (Santos Kron et al., 2020). This indicates that pyoverdine did not play a role in the biocontrol mechanism by *P. orientalis* F9. Another function of siderophores on the phyllosphere was demonstrated by Ruiz et al. (2015). The siderophores pyoverdine and enantio-pyochelin, synthetized by *P. protegens*, were responsible for its resistance against the mycotoxin fusaric acid. Fusaric acid is produced by pathogenic fungi of the *Fusarium* genus and is toxic to plants and bacteria through mechanisms that are not yet fully understood. Finally, Burbank et al. (2015) showed that mutations in the *iuc*A and *iut*A genes, responsible for siderophore and receptor biosynthesis respectively, results in a loss of surface motility of the xylem-dwelling pathogen *Pantoea stewartia*, and reduced virulence in sweet corn. This indicates that siderophores also play a role in adaptation by mediating motility. However, this mechanism has not been described yet as an adaptation strategy for phyllosphere biocontrol agents.

## Indirect Interactions

Next to direct interactions, biocontrol agents can inhibit pathogens indirectly, by modulating the plant’s immune system or hormone levels (Figure 1). Microbe-plant interactions that protect the plant against pathogen infection are discussed here as indirect interactions.

**FIGURE 2:**
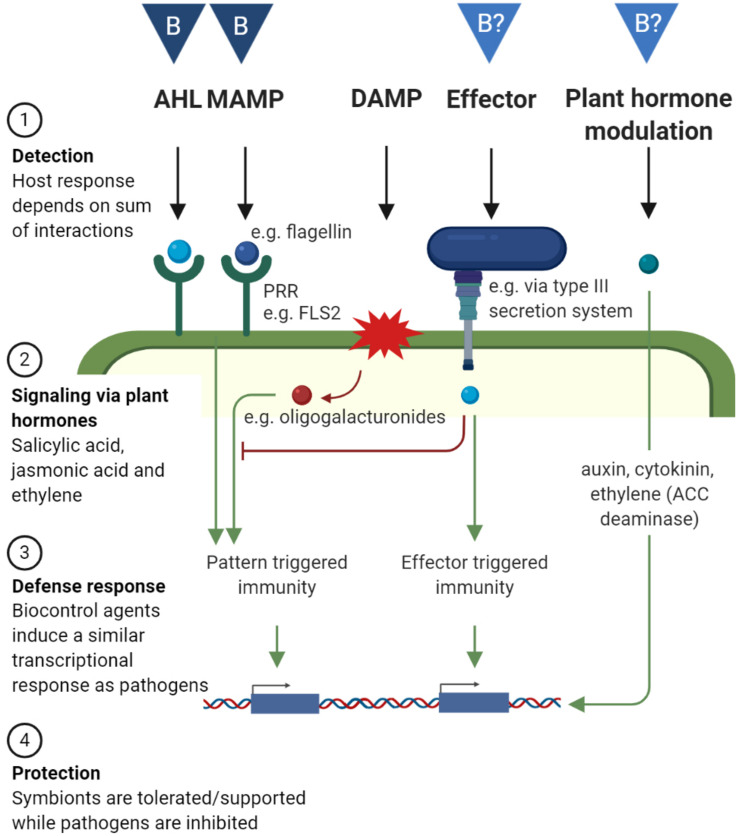
The plant’s defense system and how biocontrol agents can interact with it. The plant’s immune system can be divided in four steps: (1) Detection: A micro-organism can be detected via N-acyl homoserine lactone (AHL), microbial associated molecular patterns (MAMPs) via pattern-recognition receptors (PRRs), damage associated molecular patterns (DAMPs), effectors or other ligands which are detected intracellularly. (2) Signalization via the plant hormones salicylic acid, jasmonic acid and ethylene. (3) A defense response via changes in gene expression, and (4) Protection against the pathogen, while symbionts are tolerated or supported. Micro-organisms can also modulate plant hormone levels by producing or degrading them, which induces physiological changes in the host plant. “B” in dark blue triangles indicate known biocontrol mechanisms by which biocontrol organisms interact with the host plant (AHL and MAMP), inducing protection against pathogens. “B?” in light blue triangles indicate mechanisms for which their role in biocontrol on the phyllosphere requires confirmation (effector and plant hormone modulation). Created with BioRender.com.

Plants have evolved a complex immune system to prevent infection by recognizing potential intruders and responding with an appropriate defense response. Reversely, pathogens evolve continuously to evade recognition or to interfere with the defense response. This action and counteraction are described by Jones and Dangl (2006) in the “zigzag model.” A schematic representation of the host immune system as well as mechanisms by which biocontrol agents can interact with it is given in Figure 2. The host plant recognizes microbe-associated molecular patterns (MAMPs) by specific pattern-recognition receptors (PRRs), which leads to pattern-triggered immunity. One of the best studied MAMPs is flagellin, more specifically the epitope flg22, which is recognized by the PRR FLS2. Other MAMPs are lipopolysaccharides from Gram-negative bacteria and N-acetylglucosamine-containing glycans, such as bacterial peptidoglycan, generally more prominently in Gram-positive bacteria, fungal chitin, or rhizobacterial nodulation factors. Also volatile compounds emitted by beneficial bacteria such as *Bacillus* and *Pseudomonas* spp. can trigger the plant’s immune system, however the receptors remain to be identified (Tyagi et al., 2018). An overview of PRRs, the specific MAMPs that are recognized, and the molecular basis of the following pattern triggered immunity has been reviewed by Saijo et al. (2018). Of importance here, both pathogens and mutualistic microbes are detected through MAMP–PRR interactions and detection generally leads to relatively weak immune responses. Hacquard et al. (2017) argues that the pattern-triggered immunity does not discriminate between a beneficial or pathogenic attack, but mainly functions by restricting the microbial load. The immune response can become stronger when additional virulence factors are present, such as tissue damage or plant hormones modulation (discussed further in this section) (Jones and Dangl, 2006; Hacquard et al., 2017).

Microbes can overcome this first line of defense by modifying MAMPs or by secreting effectors into the cytoplasm of host cells that interfere with the triggered immune signaling. Consequently, plants have evolved additional mechanisms to detect these microbial effectors: effector-triggered immunity (Jones and Dangl, 2006; Figure 2). Gram-negative bacteria use type III secretion systems to deliver effector molecules into the cytoplasm to suppress the immune system. When such a secretion system is inactivated (through mutations in *hrp* genes, which are required for a functional type III secretion system and elicitation of a hypersensitive response in plants) in pathogens, disease symptoms are reduced (Hanemian et al., 2013). Such mutants are then unable to overcome pattern-triggered immunity and are unable to infect host tissue. These mutants often reside in the apoplast without causing harm and can even protect the host against invasion of the wild-type pathogen (Hanemian et al., 2013). Mutants and wild-type pathogens colonize separate cells/niches when co-inoculated. In some studies, co-inoculation led to protection (Faize et al., 2006), while in others, it was necessary to inoculate the non-virulent mutant prior to the pathogen (Feng et al., 2012). Therefore, it is debatable whether competition for nutrients and space is an important mode of action. However, multiple studies show that inoculation with *hrp* mutants induces changes in gene expression which lead to a defense response and increased resistance against the pathogen (Faize et al., 2006; Feng et al., 2012; Hanemian et al., 2013).

Type III secretion systems and effectors have mostly been described in pathogens. However, some commensals also interact more actively with the plant’s immune response via effectors. For example, nodulating rhizobacteria secrete effectors using type III and type VI secretion systems to activate nodulation in the host plant (Deakin and Broughton, 2009). Recently, Stringlis et al. (2019) identified type III secretion system gene clusters in beneficial rhizosphere *Pseudomonas* spp. These gene clusters were highly similar to type III secretion systems in other beneficial bacteria, but distinct compared to phytopathogenic *P. syringae*. Also on the phyllosphere, type III secretion systems have been identified in the genome of a non-pathogenic *Paraburkholderia isolate* (Herpell et al., 2020). However, more research is needed to determine the presence of type III secretion systems in other beneficial phyllosphere bacteria, to identify effectors associated with pathogens versus biocontrol effectors, and to determine the role of these type III secretion systems in beneficial host–microbe interactions.

A group of molecules often forgotten that have an effect on the host immune system are N-acyl-homoserine lactones (AHLs) (Schenk and Schikora, 2015). AHLs regulate the behavior of Gram-negative pathogens (see section “Quorum Sensing and Quenching”). The host plant could benefit from the ability to respond to or interfere with this quorum sensing signal. Indeed, exposing roots to AHLs or AHL-producing bacteria has been shown to trigger the upregulation of defense genes in the plant and inducing systemic resistance via salicylic acid (SA) signaling (described further) (Schenk and Schikora, 2015). One of the AHL-induced defense reactions of *Arabidopsis* plants was stomatal closure, a common first defense reaction to limit the entry of pathogens in the apoplast. Furthermore, plants react by degrading the AHLs (Delalande et al., 2005) or transporting the AHLs into the vascular system to remove them from the bacteria that produced them (Sieper et al., 2014). Both actions could be described as a plant’s equivalent to quorum quenching. Detection of AHLs in *Arabidopsis* plants is mediated through the G-protein coupled receptor encoded by *AtGPA1*. However, more research is needed to identify AHL receptors in other plants.

Finally, the plant’s immune system can be triggered by the detection of host-derived danger-associated molecular patterns (DAMPs), such as oligogalacturonides which are released from the plant cell wall during pathogen invasion. Detection of DAMPs in combination with pattern or effector triggered immunity, will result in a more severe immune response against the invader (Hacquard et al., 2017). It is unlikely biocontrol agents trigger the host immune system through the release of DAMPs.

As described here, both biocontrol as pathogenic microorganisms interact with the host immune system. Similarly to pathogen and commensal host interactions in humans, the final host response depends on the sum of the interactions with host receptors. Commensal bacteria do not trigger a strong defense reaction in the host as they lack additional virulence factors, such as triggering the formation of DAMPs, modulating plant hormone levels or secreting effectors into the host cells (Lebeer et al., 2010).

### Plant Hormones

The recognition of beneficial or pathogenic microbial attacks, as described above, leads to the activation of signaling hormones in the plant, as counterparts of immune modulating cytokines in human and animal cells. Relevant plant hormones include SA, JA, and ethylene, where SA and JA are considered to act antagonistic (Koornneef and Pieterse, 2008; Pieterse et al., 2014). JA and ethylene are usually involved in the defense response against necrotrophic pathogens (feeding on killed host cells), or after wounding, while SA is involved in the defense reaction against biotrophic or hemibiotrophic pathogens (feeding on living tissue) (Glazebrook, 2005). Experiments spraying bacterial produced rhamnolipids on leaves of Arabidopsis (see section “Direct Interactions”) showed that SA plays a central role in rhamnolipid-mediated disease resistance (Sanchez et al., 2012).

A first example on how phyllosphere microbes can directly modulate plant hormone levels, is through the enzyme 1-aminocyclopropane-1-carboxylate (ACC) deaminase that degrades the ethylene precursor ACC. It has been detected in plant-growth promoting rhizosphere bacteria such as *Azospirillum*, *Rhizobium*, and *Pseudomonas* spp. (Gamalero and Glick, 2015; Nascimento et al., 2019), as well as in phyllosphere bacteria, such as several *Methylobacterium* spp. (Kwak et al., 2014) and *Rhodococcus fascians* (Chinnadurai et al., 2009; Francis et al., 2016). 1-Aminocyclopropane-1-carboxylate deaminase activity lowers ethylene levels, reducing the plant’s defense responses and thereby facilitating symbiotic microorganisms. ACC deaminase also results in the promotion of plant growth, since plants become more resilient against environmental stress such as drought, flooding, salt stress or pathogen pressure (Gamalero and Glick, 2015; Nascimento et al., 2018; Saghafi et al., 2020). Direct evidence for a role of ethylene in modulating the community composition of the phyllosphere is given by Bodenhausen et al. (2014), as ethylene-insensitive plant mutants harbored a different phyllosphere community. Moreover, evidence is rising for a direct role of ACC in regulating plant development (Van de Poel and Van Der Straeten, 2014; Vanderstraeten and van Der Straeten, 2017) and defense responses (Tsang et al., 2011).

Levels of phytohormones that are primarily involved in plant growth, such as cytokinins and auxins, can also be modulated by microbes (Leach et al., 2017). Both production and degradation of the auxin indole-3-acetic acid (IAA) have been observed in both plant growth-promoting and pathogenic bacteria (Duca et al., 2014; Nascimento et al., 2019). Degradation of IAA can be advantageous for phyllosphere microbes in two ways. On the one hand, IAA is a good source of carbon and nitrogen (Leveau and Lindow, 2005; Nascimento et al., 2019). On the other hand, manipulation of IAA levels induces physiological changes in the plant, such as cell wall-loosening and the release of nutrients that benefit the survival or colonization of the microbe (Vanderhoef and Dute, 1981). *P. putida* 1290 is able to grow on IAA as a sole source of carbon, nitrogen, and energy (Leveau and Lindow, 2005). This ability of *P. putida* 1290 is encoded by the *iac* gene cluster. Homologs of the *iac* gene cluster have been identified in strains from various genera, such as *P. putida* GB-1, *Marinomonas* sp. MWYL1, *Burkholderia* sp. 383, *Sphingomonas wittichii* RW1, *Rhodococcus* sp. RHA, *Acinetobacter baumannii* ATCC 19606, and *Lelliottia* sp. (Leveau and Gerards, 2008; Lin et al., 2012). On the other hand, high levels of IAA, produced by plant or bacterium, can play an important role in disease development [e.g., by gall forming pathogens *Rhodococcus fascians* (Stes et al., 2012) (see further in text), knot development by *P. savastanoi* (Surico et al., 1985) or suppression of the host defense system by *P. syringae* pv. *syringae* DC3000 (McClerklin et al., 2018)]. Bacterial degradation of IAA has so far not been directly linked with antipathogenic effects. However, IAA degradation is an important adaptation mechanism of bacteria on the phyllosphere.

Besides degradation, IAA can also be produced by plant-associated bacteria. Production of IAA can occur via several pathways, as reviewed by Duca et al. (2014). The presence of these pathways can be detected by the presence of the following essential genes: *ipd*C and *ald*H for the indole-3-pyruvate pathway (encoding the enzymes necessary for the decarboxylation of indole-3-pyruvate and subsequent oxidation, respectively), *dcc* and *ald*H for the tryptamine pathway (encoding the enzymes necessary for the decarboxylation of tryptophan and subsequent oxidation, respectively), *iaa*M and *iaa*H for the indole-3-acetamide pathway (encoding for tryptophan-2-monooxygenase and indole-3-acetamide hydrolase, respectively), and *nth*A for the indole-3-acetonitrile pathway (encoding for nitrile hydratase α) (de Souza et al., 2019). It is important to note that although the indole-3-acetamide pathway was considered as being exclusive for the excessive IAA production by gall forming bacteria like *P. savastanoi*, *Erwinia* spp., and *Agrobacterium* transformed plant tissue (Jameson, 2000), these genes are also present in methylotrophic rhizosphere microorganisms (Li et al., 2019). The *ipd*C gene is of special interest since it was demonstrated that the specific growth conditions in the phyllosphere trigger the expression of the *ipd*C gene in symbiotic *P. agglomerans* (syn. *E. herbicola*) (Brandl et al., 2001). Furthermore, the phyllosphere consists of microenvironments that induce differential expression of the *ipd*C gene. Expression of the *ipd*C gene and production of IAA is induced in the rhizosphere symbiont *Azospirillum brasilense* Sp245 when carbon source availability is limited in batch and fed-batch cultures (Ona et al., 2005). These observations indicate that IAA production, encoded on the *ipd*C gene, is used by beneficial plant-associated bacteria to adapt to the phyllosphere by improving their growth conditions and availability of carbon sources. However, as with IAA degradation, IAA production is an important adaptation factor and has not yet been linked to biocontrol activity.

Members of the genus *Methylobacterium* enhance plant growth by producing auxins and cytokinins (Koenig et al., 2002; Kwak et al., 2014; Jorge et al., 2019; Li et al., 2019). Interaction with the host is beneficial for the symbiont’s growth since they metabolize the methanol released as the plant grows (Kutschera, 2007). *Methylobacterium* derived cytokinins were attributed to drought/saline stress resistance in the host (Jorge et al., 2019). Both the type of cytokinins present and the presence of a *mia*A gene indicate that methylobacterial cytokinin production is merely via tRNA (Koenig et al., 2002; Kwak et al., 2014; Jorge et al., 2019). Moreover, biocontrol activity has been observed, for example, seed inoculation of groundnut plants with *Methylobacterium* spp. increased protection against pathogens *Aspergillus niger* and *Sclerotium rolfsii* (Madhaiyan et al., 2006). The treatment with *Methylobacterium* spp. induced an increased activity of enzymes in the host plant that are typically associated with Induced Systemic Resistance (ISR) a state in which the plant’s immune system is triggered in order to become resistant against pathogen infection (see further for the paragraph on Induced Systemic Responses). This indicates that the applied *Methylobacterium* spp. interacted with the host plant’s defense system resulting in protection against *A. niger* and *S. rolfsii.* However, the specific role of microbial production of cytokinins and auxins in the plant’s defense response has not been elucidated in this study.

Bacteria that are known to modulate plant hormone levels are *Rhodococcus fascians.* Both pathogenic as non-pathogenic *R. fascians* have the ability to both produce IAA and cytokinins, and decrease ethylene levels (Francis et al., 2016). In pathogenic bacteria, the genes encoding auxin and cytokinin production are plasmid-borne (Jameson, 2000). IAA production is higher in presence of exogenous tryptophan, a precursor of IAA. Interestingly, upon inoculation of the plant with pathogenic or non-pathogenic *R. fascians*, the metabolism of the host plant changes and more tryptophan is accumulated, possibly stimulating bacterial production of IAA (Francis et al., 2016). On the other hand, the production of cytokinins by *R. fascians* induce increased auxin production in the plant. The increased auxin levels play an important role in the development of disease symptoms (Stes et al., 2012). The non-pathogenic derivative of this strain lacks the plasmid with virulence genes. The main pathogenicity factor on the plasmid is the production of modified methylated cytokinins, which are not degraded by cytokinin oxidase activity, mimic plant cytokinins, induces increased auxin production in plants and results in the development of disease symptoms (Radhika et al., 2015). Cytokinin and auxin production in pathogenic *R. fascians* is thus detrimental and contributes to the disease development. Reversely, cytokinin and auxin production in non-pathogenic *R. fascians*, as well as in other symbionts (e.g., *Methylobacteria*, described above), is being described as a beneficial trait since it promotes plant growth (Schauer and Kutschera, 2011; Francis et al., 2016; Romero et al., 2016).

In conclusion we can postulate that, through the ability to control the auxin steady state by producing additional auxins on the one hand, and by auxin degradation when excessive auxin production occurs in case an additional pathogen is invading the host on the other hand, a benign symbiont is possibly able to optimize its ecological niche both by improving the host growth and excluding other invaders. The cross talk between auxins and ethylene, as well as the bacterial ACC-degradation might prevent ethylene induced excessive immune and senescence responses to occur. Moreover, it is of general knowledge that cytokinins act as a sink for sugar and other metabolites (Roitsch and Ehneß, 2000), therefore we postulate that it is plausible that the microbial cytokinin production works as a sink for metabolites to the benefit of the symbiont (carbon source) as well as the host by reducing senescence and as a consequence prolonging photosynthetic activities.

### Induced Systemic Responses

Once a microbe is detected by the plant and its presence is signaled via plant hormones, as described above, a specific immune response is triggered in the plant (Fig 2). Beneficial microbes are able to trigger the plant’s defense system at the point of recognition and induce immunity against pathogens in the whole plant body (Pieterse et al., 2014). This phenomenon is called induced systemic resistance (ISR). Also pathogens can induce a systemic response, which then protects other parts of the plants, this is called systemic acquired resistance (SAR). The term ISR is usually used when it is triggered by rhizosphere microbes (Pieterse et al., 2014). However, in the next paragraphs, we will describe several examples of ISR by beneficial microorganisms in the phyllosphere. Also endophytic bacteria can trigger ISR (Kloepper and Ryu, 2007).

Vogel et al. (2016) studied the differences in gene expression in *Arabidopsis* plants upon inoculation with two model commensal phyllosphere bacteria; *S. melonis* Fr1 and *Methylobacterium extorquens* PA1. Colonization by *M. extorquens* PA1 resulted in very little transcriptional response from the plant whereas *S. melonis* Fr1 changed the expression of several hundreds of genes. This corresponds with the findings of Innerebner et al. (2011) where *S. melonis* Fr1 decreased disease development on *A. thaliana* while *M. extorquens* PA1 did not. The transcriptional response induced by *S. melonis* Fr1 was similar to the response induced by an encounter with the pathogenic *P. syringae* DC3000 (Vogel et al., 2016). The authors hypothesize that plants detect the presence of *S. melonis* Fr1 in a similar way as *P. syringae* and respond with an expression of defense-related genes that are involved in plant protection. However, the plant’s response is less severe, probably because *S. melonis Fr1* lacks additional virulence factors which are present in *P. syringae* DC3000. The exact mechanisms still need to be unraveled, since plant mutants defective in several known defense signaling pathways, such as SA and JA signaling, and lacking the FLS2 PRR, showed that these pathways and receptors where not involved. More recently, Ryffel et al. (2016) demonstrated that *S. melonis* Fr1 induced camalexin production in the host plant. The pathogen *P. syringae* pv. *tomato* also induced production of camalexin, yet in higher concentrations. Production of the tryptophan-derived indole alkaloid camalexin, is a typical defense response of *Arabidopsis* and other plants from the Crucifereae family. Due to its lipophilic nature, camalexin is effective against a wide range of bacteria and fungi by interfering with the integrity of membranes (e.g., by binding to phospholipids). Production of camalexin by *Arabidopsis*, triggered by *S. melonis* Fr1 is thus postulated to be the mechanism behind the observed plant protection by this commensal.

The host’s immune system does not only target bacterial or fungal pathogens, but can also protect against viral diseases (Lee and Ryu, 2016). Three-year field trials of foliar applications of *Bacillus amyloliquefasciens* 5B6 showed consistently reduced cucumber mosaic virus accumulation as measured by qPCR (Lee and Ryu, 2016). Observed changes in gene expression in the host plant suggested that activation of SA and ethylene signaling pathways played a key role in the acquired resistance. Also here, the genes upregulated by the biocontrol agent were the same as the genes upregulated in the defense response caused by several viruses, including cucumber mosaic virus (Park et al., 2004). *B. amyloliquefasciens* 5B6 colonized the phyllosphere successfully as their population size remained stable during seven days after administration of 10^8^ CFU/ml until run-off. This contrasted with the sharp decline in population size of strain FZB42, isolated from the soil, showing that strain-specific adaptation traits are important for survival and successful biocontrol in the phyllosphere.

Another intriguing observation was made by Hong et al. (2016) on the known plant-growth promoting rhizosphere bacterium *Paenibacillus polymyxa* AC-1. This strain inhibited the growth of phyllosphere pathogens *P. syringae* pv. *tomato* DC3000 and *P. syringae* pv. *tabaci* in an *in vitro* setting. Cell-free supernatant of *P. polymyxa* AC-1 also suppressed these pathogens, suggesting that antimicrobial metabolites excreted by the antagonist play a direct antagonistic role (see section “Antimicrobial Metabolites”). Inoculation of the root tips of axenic *Arabidopsis* seedlings with bacterial suspensions of *P. polymyxa* AC-1 resulted in a SA and JA-dependent defense reaction. Interestingly, this inoculation of the roots of axenic plants resulted in colonization of the *Arabidopsis* leaf endosphere with *P. polymyxa* AC-1. Colonization of the leaf endosphere was 10-fold higher in *Arabidopsis* mutants with reduced sensitivity to JA and 10-fold lower in mutants deficient in the isoprenoid plant hormone abscisic acid, compared to wild-type plants. The colonization of the leaf endosphere by *P. polymyxa* AC-1 in JA-deficient plants even caused disease symptoms in the phyllopshere. This indicates that JA negatively impacts the detrimental endophytic growth of AC-1. This illustrates that the plant’s defense system is important in regulating the total microbial load and preventing symbiotic bacteria to become invasive.

The mechanisms by which microbes are detected by the host and subsequently trigger the host’s immune response, are similar in both non-pathogenic and pathogenic strains (Fig 2). However, non-pathogenic strains lack additional virulence factors, resulting in a milder defense response from the host. Biocontrol agents have the ability to trigger the immune system, and thereby inducing resistance to phylogenetically distinct pathogens. Sometimes, biocontrol agents are very closely related to pathogenic strains, or can even be opportunistic pathogens themselves (e.g., *Pseudomonas*, *R. fascians, P. polymyxa*), in this case the difference between ISR and SAR becomes less clear.

## Conclusion and Future Research Perspectives

The phyllosphere harbors a diverse set of microbes. These microbes interact closely with each other and with the host plant. Amongst them are pathogens, causing disease in the host plant and reducing yields in agriculture, but also beneficial microbes which can be the key to environmentally friendly solutions to protect crops from diseases. These beneficial microbes can inhibit pathogen growth directly, by competing for nutrients and space, by interfering with their communication, by excreting antimicrobial metabolites or enzymes, or by parasitizing on the pathogen. On the other hand, beneficial microbes can trigger the plant’s immune response and modulate plant hormone levels, and hereby indirectly inhibiting pathogen growth.

Knowledge on these mechanisms is often gained through *in vitro* experiments using gnotobiotic or sterile plants, while the efficacy of a biocontrol agent needs to be validated in field trials. In this review we linked these two types of studies and gave an overview of biocontrol mechanisms and adaptation mechanisms that play a role in the phyllosphere. Several mechanisms still need further validation, for example, the characterization of novel antimicrobial peptides, the role of type III secretion systems, the biocontrol potential of small peptides involved in quorum sensing and the impact of bacterially produced plant hormones on the host immune system. Next, we need to understand which mechanisms are at play in field conditions. This question has also been addressed by Köhl et al. (2019). Firstly, which biocontrol mechanisms are active on the field, e.g., some antimicrobial metabolites play a role in biocontrol *in vitro* but not *in vivo*, and reversely (Köhl et al., 2019; Santos Kron et al., 2020)? Secondly, is the biocontrol agent adapted to the phyllosphere in field conditions, and can it disperse in the growing plant (e.g., Wei et al., 2016)? Finally, the biocontrol agent needs to integrate in the resident microbial community. This resident community can positively or negatively influence the biocontrol activity and the success of colonization of the biocontrol agent (Massart et al., 2015).

Techniques that can help us understand the mechanisms playing a role in complex phyllosphere communities are metagenome, metatranscriptome, metaproteome, and metabolome analyses. Eventually, this understanding may allow us to go beyond the application of single strains, and toward designing communities, an idea that is often repeated in biocontrol research (Massart et al., 2015; Singh and Trivedi, 2017). Biocontrol communities possess a variety of complementary adaptation and biocontrol factors, that co-operate in suppressing the disease and surviving on the phyllosphere. Biocontrol agents and communities can be an effective and sustainable alternative to conventional pesticides, which is needed to safeguard our crop production.

## Author Contributions

ML and SL conceived and designed the manuscript. ML wrote the manuscript and prepared the figures. All authors critically reviewed the manuscript and contributed to figure improvement, with special attention toward their specific expertise ranging from phyllosphere bacterial communities, bacterial antimicrobial compounds, probiotics, bacterial genome analysis and functional prediction, plant hormones, plant immune system toward ecology in general and approved the final version of the manuscript.

## Conflict of Interest

The authors declare that the research was conducted in the absence of any commercial or financial relationships that could be construed as a potential conflict of interest.

## References

[B1] Agler M. T. Ruhe J. Kroll S. Morhenn C. Kim S. T. Weigel D. (2016). Microbial hub taxa link host and abiotic factors to plant microbiome variation. *PLoS Biol.* 14:e1002352. 10.1371/journal.pbio.1002352 26788878 PMC4720289

[B2] Ali G. S. El-Sayed A. S. A. Patel J. S. Green K. B. Ali M. Brennan M. (2016). Ex Vivo application of secreted metabolites produced by soil-inhabiting *Bacillus* spp. Efficiently controls foliar diseases caused by *Alternaria* spp. *Appl. Environ. Microbiol.* 82 478–490. 10.1128/aem.02662-15 26519395 PMC4711143

[B3] Allonsius C. N. Vandenheuvel D. Oerlemans E. F. M. Petrova M. I. Donders G. G. G. Cos P. (2019). Inhibition of *Candida albicans* morphogenesis by chitinase from Lactobacillus rhamnosus GG. *Sci. Rep.* 9:2900. 10.1038/s41598-019-39625-0 30814593 PMC6393446

[B4] Alymanesh M. R. Taheri P. Tarighi S. (2016). *Pseudomonas* as a frequent and important quorum quenching bacterium with biocontrol capability against many phytopathogens. *Biocontrol Sci. Technol.* 26 1719–1735. 10.1080/09583157.2016.1239065

[B5] Bailly A. Weisskopf L. (2017). Mining the volatilomes of plant-associated microbiota for new biocontrol solutions. *Front. Microbiol.* 8:1638. 10.3389/fmicb.2017.01638 28890716 PMC5574903

[B6] Bakker P. A. H. M. Pieterse C. M. J. Van Loon L. C. (2007). Induced Systemic Resistance by fluorescent *Pseudomonas* spp. *Phytopathology* 97 239–243. 10.1094/PHYTO-97-2-0239 18944381

[B7] Berg M. Koskella B. (2018). Nutrient- and Dose-Dependent Microbiome-Mediated Protection against a Plant Pathogen. *Curr. Biol.* 28:2487-2492.e3. 10.1016/j.cub.2018.05.085 30057302

[B8] Bernal P. Allsopp L. P. Filloux A. Llamas M. A. (2017). The *Pseudomonas putida* T6SS is a plant warden against phytopathogens. *ISME J.* 11 972–987. 10.1038/ismej.2016.169 28045455 PMC5363822

[B9] Bernal P. Llamas M. A. Filloux A. (2018). Type VI secretion systems in plant-associated bacteria. *Environ. Microbiol.* 20 1–15. 10.1111/1462-2920.13956 29027348 PMC5813230

[B10] Blanvillain S. Meyer D. Boulanger A. Lautier M. Guynet C. Denancé N. (2007). Plant carbohydrate scavenging through TonB-dependent receptors: a feature shared by phytopathogenic and aquatic bacteria. *PLoS One* 2:e224. 10.1371/journal.pone.0000224 17311090 PMC1790865

[B11] Blin K. Shaw S. Steinke K. Villebro R. Ziemert N. Lee S. Y. (2019). antiSMASH 5.0: updates to the secondary metabolite genome mining pipeline. *Nucleic Acids Res.* 47 W81–W87. 10.1093/nar/gkz310 31032519 PMC6602434

[B12] Bodenhausen N. Bortfeld-Miller M. Ackermann M. Vorholt J. A. (2014). A synthetic community approach reveals plant genotypes affecting the phyllosphere microbiota. *PLoS Genet.* 10:e1004283. 10.1371/journal.pgen.1004283 24743269 PMC3990490

[B13] Boller T. (1993). “Antimicrobial Functions of the Plant Hydrolases, Chitinase and ß-1,3-Glucanase,” in *Mechanisms of Plant Defense Responses. Developments in Plant Pathology*, Vol 2 Fritig B. Legrand M. (Dordrecht: Springer), 391–400. 10.1007/978-94-011-1737-1_124

[B14] Brandl M. T. Quiñones B. Lindow S. E. (2001). Heterogeneous transcription of an indoleacetic acid biosynthetic gene in Erwinia herbicola on plant surfaces. *Proc. Natl. Acad. Sci. U.S.A.* 98 3454–3459. 10.1073/pnas.061014498 11248099 PMC30674

[B15] Broeckx G. Vandenheuvel D. Claes I. J. J. Lebeer S. Kiekens F. (2016). Drying techniques of probiotic bacteria as an important step towards the development of novel pharmabiotics. *Int. J. Pharm.* 505 303–318. 10.1016/j.ijpharm.2016.04.002 27050865

[B16] Burbank L. Mohammadi M. Roper M. C. (2015). Siderophore-mediated iron acquisition influences motility and is required for full virulence of the xylem-dwelling bacterial phytopathogen *Pantoea stewartii* subsp. stewartii. *Appl. Environ. Microbiol.* 81 139–148. 10.1128/AEM.02503-14 25326304 PMC4272718

[B17] Chen W. J. Kuo T. Y. Hsieh F. C. Chen P. Y. Wang C. S. Shih Y. L. (2016). Involvement of type VI secretion system in secretion of iron chelator pyoverdine in *Pseudomonas taiwanensis*. *Sci. Rep.* 6 1–14. 10.1038/srep32950 27605490 PMC5015096

[B18] Chen X. H. Koumoutsi A. Scholz R. Eisenreich A. Schneider K. Heinemeyer I. (2007). Comparative analysis of the complete genome sequence of the plant growth-promoting bacterium *Bacillus amyloliquefaciens* FZB42. *Nat. Biotechnol.* 25 1007–1014. 10.1038/nbt1325 17704766

[B19] Chen X. H. Scholz R. Borriss M. Junge H. Mögel G. Kunz S. (2009). Difficidin and bacilysin produced by plant-associated *Bacillus amyloliquefaciens* are efficient in controlling fire blight disease. *J. Biotechnol.* 140 38–44. 10.1016/j.jbiotec.2008.10.015 19061923

[B20] Chen Y. Wang J. Yang N. Wen Z. Sun X. Chai Y. (2018). Wheat microbiome bacteria can reduce virulence of a plant pathogenic fungus by altering histone acetylation. *Nat. Commun.* 9:3429. 10.1038/s41467-018-05683-7 30143616 PMC6109063

[B21] Chin-A-Woeng T. F. C. Bloemberg G. V. Lugtenberg B. J. J. (2003). Phenazines and their role in biocontrol by *Pseudomonas* bacteria. *New Phytol.* 157 503–523. 10.1046/j.1469-8137.2003.00686.x 33873412

[B22] Chinnadurai C. Balachandar D. Sundaram S. P. (2009). Characterization of 1-aminocyclopropane-1-carboxylate deaminase producing methylobacteria from phyllosphere of rice and their role in ethylene regulation. *World J. Microbiol. Biotechnol.* 25 1403–1411. 10.1007/s11274-009-0027-1

[B23] Van de Poel B. Van Der Straeten D. (2014). 1-aminocyclopropane-1-carboxylic acid (ACC) in plants: more than just the precursor of ethylene!. *Front. Plant Sci.* 5:640. 10.3389/fpls.2014.00640 25426135 PMC4227472

[B24] de Souza R. S. C. Armanhi J. S. L. Damasceno N. de B. Imperial J. Arruda P. (2019). Genome sequences of a plant beneficial synthetic bacterial community reveal genetic features for successful plant colonization. *Front. Microbiol.* 10:1779. 10.3389/fmicb.2019.01779 31456759 PMC6701196

[B25] Deakin W. J. Broughton W. J. (2009). Symbiotic use of pathogenic strategies: rhizobial protein secretion systems. *Nat. Rev. Microbiol.* 7 312–320. 10.1038/nrmicro2091 19270720

[B26] Delalande L. Faure D. Raffoux A. Uroz S. D’Angelo-Picard C. Elasri M. (2005). N-hexanoyl-L-homoserine lactone, a mediator of bacterial quorum-sensing regulation, exhibits plant-dependent stability and may be inactivated by germinating Lotus corniculatus seedlings. *FEMS Microbiol. Ecol.* 52 13–20. 10.1016/j.femsec.2004.10.005 16329888

[B27] Delmotte N. Claudia K. Samuel C. Gerd I. Bernd R. Ralph S. (2009). Community proteogenomics reveals insights into the physiology of phyllosphere bacteria. *Proc. Natl. Acad. Sci. U.S.A.* 106 16428–16433. 10.1073/pnas.0905240106 19805315 PMC2738620

[B28] Duca D. Lorv J. Patten C. L. Rose D. Glick B. R. (2014). Indole-3-acetic acid in plant-microbe interactions. *Antonie van Leeuwenhoek* 106 85–125. 10.1007/s10482-013-0095-y 24445491

[B29] Dunlap C. A. Bowman M. J. Schisler D. A. (2013). Genomic analysis and secondary metabolite production in *Bacillus amyloliquefaciens* AS 43.3: a biocontrol antagonist of Fusarium head blight. *Biol. Control* 64 166–175. 10.1016/j.biocontrol.2012.11.002

[B30] Edwards S. G. Seddon B. (2001). Mode of antagonism of Brevibacillus brevis against Botrytis cinerea in vitro. *J. Appl. Microbiol.* 91 652–659. 10.1046/j.1365-2672.2001.01430.x 11576302

[B31] Essghaier B. Hedi A. Halaoui M. R. Boudabous A. Sadfi-Zouaoui N. (2012). In vivo and in vitro evaluation of antifungal activities from a halotolerant Bacillus subtilis strain J9. *Afr. J. Microbiol. Res.* 6 4073–4083. 10.5897/ajmr11.403

[B32] Faize M. Brisset M. N. Perino C. Vian B. Barny M. A. Paulin J. P. (2006). Protection of apple against fire blight induced by an hrpL mutant of *Erwinia amylovora*. *Biol. Plant.* 50 667–674. 10.1007/s10535-006-0104-3

[B33] Feng D. X. Tasset C. Hanemian M. Barlet X. Hu J. Trémousaygue D. (2012). Biological control of bacterial wilt in Arabidopsis thaliana involves abscissic acid signalling. *New Phytol.* 194 1035–1045. 10.1111/j.1469-8137.2012.04113.x 22432714

[B34] Fernando W. G. D. Nakkeeran S. Zhang Y. Savchuk S. (2007). Biological control of *Sclerotinia sclerotiorum* (Lib.) de Bary by *Pseudomonas* and *Bacillus* species on canola petals. *Crop Prot.* 26 100–107. 10.1016/j.cropro.2006.04.007

[B35] Francis I. M. Stes E. Zhang Y. Rangel D. Audenaert K. Vereecke D. (2016). Mining the genome of *Rhodococcus fascians*, a plant growth-promoting bacterium gone astray. *N. Biotechnol.* 33 706–717. 10.1016/j.nbt.2016.01.009 26877150

[B36] Gamalero E. Glick B. R. (2015). Bacterial modulation of plant ethylene levels. *Plant Physiol.* 169 13–22. 10.1104/pp.15.00284 25897004 PMC4577377

[B37] Giddens S. R. Feng Y. Mahanty H. K. (2002). Characterization of a novel phenazine antibiotic gene cluster in *Erwinia herbicola* Eh1087. *Mol. Microbiol.* 45 769–783. 10.1046/j.1365-2958.2002.03048.x 12139622

[B38] Giddens S. R. Houliston G. J. Mahanty H. K. (2003). The influence of antibiotic production and pre-emptive colonization on the population dynamics of Pantoea agglomerans (Erwinia herbicola) Eh1087 and *Erwinia amylovora* in planta. *Environ. Microbiol.* 5 1016–1021. 10.1046/j.1462-2920.2003.00506.x 14510856

[B39] Glazebrook J. (2005). Contrasting mechanisms of defense against biotrophic and necrotrophic pathogens. *Annu. Rev. Phytopathol.* 43 205–227. 10.1146/annurev.phyto.43.040204.135923 16078883

[B40] Guennoc C. M. Rose C. Labbé J. Deveau A. (2018). Bacterial biofilm formation on the hyphae of ectomycorrhizal fungi: a widespread ability under controls? *FEMS Microbiol. Ecol.* 94:fiy093. 10.1093/femsec/fiy093 29788056

[B41] Hacquard S. Spaepen S. Garrido-Oter R. Schulze-Lefert P. (2017). Interplay between innate immunity and the plant microbiota. *Annu. Rev. Phytopathol.* 55 565–589. 10.1146/annurev-phyto-080516-35623 28645232

[B42] Haefele D. M. Lindow S. E. (1987). Flagellar Motility Confers Epiphytic Fitness Advantages upon *Pseudomonas syringae*. *Appl. Environ. Microbiol.* 53 2528–2533. 10.1128/aem.53.10.2528-2533.1987 16347469 PMC204140

[B43] Hanemian M. Zhou B. Deslandes L. Marco Y. Trémousaygue D. (2013). Hrp mutant bacteria as biocontrol agents: toward a sustainable approach in the fight against plant pathogenic bacteria. *Plant Signal. Behav.* 8:e25678. 10.4161/psb.25678 23887499 PMC4091062

[B44] Helfrich E. J. N. N. Vogel C. M. Ueoka R. Schäfer M. Ryffel F. Müller D. B. (2018). Bipartite interactions, antibiotic production and biosynthetic potential of the Arabidopsis leaf microbiome. *Nat. Microbiol.* 3 909–919. 10.1038/s41564-018-0200-0 30038309 PMC7115891

[B45] Hernandez M. N. Lindow S. E. (2019). *Pseudomonas syringae* increases water availability in leaf microenvironments via production of hygroscopic syringafactin. *Appl. Environ. Microbiol.* 85 e1014–e1019. 10.1128/AEM.01014-19 31285194 PMC6715840

[B46] Herpell J. B. Schindler F. Bejtoviæ M. Fragner L. Diallo B. Bellaire A. (2020). The Potato Yam Phyllosphere Ectosymbiont *Paraburkholderia* sp. Msb3 is a potent growth promotor in tomato. *Front. Microbiol.* 11:581. 10.3389/fmicb.2020.00581 32373084 PMC7186400

[B47] Hill C. Guarner F. Reid G. Gibson G. R. Merenstein D. J. Pot B. (2014). Expert consensus document: the international scientific association for probiotics and prebiotics consensus statement on the scope and appropriate use of the term probiotic. *Nat. Rev. Gastroenterol. Hepatol.* 11 506–514. 10.1038/nrgastro.2014.66 24912386

[B48] Hong C. E. Kwon S. Y. Park J. M. (2016). Biocontrol activity of Paenibacillus polymyxa AC-1 against *Pseudomonas syringae* and its interaction with *Arabidopsis thaliana*. *Microbiol. Res.* 185 13–21. 10.1016/j.micres.2016.01.004 26946374

[B49] Hosni T. Moretti C. Devescovi G. Suarez-Moreno Z. R. Fatmi M. B. Guarnaccia C. (2011). Sharing of quorum-sensing signals and role of interspecies communities in a bacterial plant disease. *ISME J.* 5 1857–1870. 10.1038/ismej.2011.65 21677694 PMC3223305

[B50] Innerebner G. Knief C. Vorholt J. A. (2011). Protection of Arabidopsis thaliana against leaf-pathogenic *Pseudomonas syringae* by *Sphingomonas* strains in a controlled model system. *Appl. Environ. Microbiol.* 77 3202–3210. 10.1128/AEM.00133-11 21421777 PMC3126462

[B51] Jameson P. E. (2000). “Cytokinins and auxins in plant-pathogen interactions - An overview,” in *Plant Growth Regulation*, Ed. Chen Z.-H. (Berlin: Springer), 369–380. 10.1023/A:1010733617543

[B52] Ji P. Wilson M. (2002). Assessment of the importance of similarity in carbon source utilization profiles between the biological control agent and the pathogen in biological control of bacterial speck of tomato. *Appl. Environ. Microbiol.* 68 4383–4389. 10.1128/AEM.68.9.4383-4389.2002 12200291 PMC124063

[B53] Jones J. D. G. Dangl J. L. (2006). The plant immune system. *Nature* 444 323–329. 10.1038/nature05286 17108957

[B54] Jorge G. L. Kisiala A. Morrison E. Aoki M. Nogueira A. P. O. Emery R. J. N. (2019). Endosymbiotic *Methylobacterium oryzae* mitigates the impact of limited water availability in lentil (*Lens culinaris* Medik.) by increasing plant cytokinin levels. *Environ. Exp. Bot.* 162 525–540. 10.1016/j.envexpbot.2019.03.028

[B55] Kamber T. Lansdell T. A. Stockwell V. O. Ishimaru C. A. Smits T. H. M. Duffy B. (2012). Characterization of the biosynthetic operon for the antibacterial peptide herbicolin in *Pantoea vagans* biocontrol strain C9-1 and incidence in *Pantoea* species. *Appl. Environ. Microbiol.* 78 4412–4419. 10.1128/AEM.07351-11 22504810 PMC3370561

[B56] Kim B.-Y. Lee S.-Y. Ahn J.-H. Song J. Kim W.-G. Weon H.-Y. (2015). Complete Genome Sequence of *Bacillus amyloliquefaciens* subsp. plantarum CC178, a Phyllosphere Bacterium Antagonistic to Plant Pathogenic Fungi. *Genome Announc.* 3:e01368-14. 10.1128/genomeA.01368-14 25573933 PMC4290986

[B57] Kleerebezem M. (2004). Quorum sensing control of lantibiotic production; nisin and subtilin autoregulate their own biosynthesis. *Peptides* 25 1405–1414. 10.1016/j.peptides.2003.10.021 15374644

[B58] Kloepper J. W. Ryu C.-M. (2007). “Bacterial endophytes as elicitors of induced systemic resistance,” in *Microbial Root Endophytes*, eds Boyle C. J.C. Sieber T. N. Schulz B. J. E. (Berlin: Springer Berlin Heidelberg), 33–52. 10.1007/3-540-33526-9_3

[B59] Koenig R. L. Morris R. O. Polacco J. C. (2002). tRNA is the source of low-level trans-zeatin production in *Methylobacterium* spp. *J. Bacteriol.* 184 1832–1842. 10.1128/JB.184.7.1832-1842.2002 11889088 PMC134930

[B60] Köhl J. Kolnaar R. Ravensberg W. J. (2019). Mode of action of microbial biological control agents against plant diseases: relevance beyond efficacy. *Front. Plant Sci.* 10:845. 10.3389/fpls.2019.00845 31379891 PMC6658832

[B61] Koornneef A. Pieterse C. M. J. (2008). Cross talk in defense signaling. *Plant Physiol.* 146 839–844. 10.1104/pp.107.112029 18316638 PMC2259093

[B62] Kramer J. Özkaya Ö Kümmerli R. (2019). Bacterial siderophores in community and host interactions. *Nat. Rev. Microbiol.* 18 152–163. 10.1038/s41579-019-0284-4 31748738 PMC7116523

[B63] Kutschera U. (2007). Plant-associated methylobacteria as co-evolved phytosymbionts: a hypothesis. *Plant Signal. Behav.* 2 74–78. 10.4161/psb.2.2.4073 19516971 PMC2633902

[B64] Kwak M. J. Jeong H. Madhaiyan M. Lee Y. Sa T. M. Oh T. K. (2014). Genome information of *Methylobacterium oryzae*, a plant-probiotic methylotroph in the phyllosphere. *PLoS One* 9:e106704. 10.1371/journal.pone.0106704 25211235 PMC4161386

[B65] Laforest-Lapointe I. Messier C. Kembel S. W. (2016). Host species identity, site and time drive temperate tree phyllosphere bacterial community structure. *Microbiome* 4:27. 10.1186/s40168-016-0174-1 27316353 PMC4912770

[B66] Leach J. E. Triplett L. R. Argueso C. T. Trivedi P. (2017). Communication in the Phytobiome. *Cell* 169 587–596. 10.1016/j.cell.2017.04.025 28475891

[B67] Lebeer S. Vanderleyden J. De Keersmaecker S. C. J. (2008). Genes and molecules of *Lactobacilli* supporting probiotic action. *Microbiol. Mol. Biol. Rev.* 72 728–764. 10.1128/mmbr.00017-08 19052326 PMC2593565

[B68] Lebeer S. Vanderleyden J. De Keersmaecker S. C. J. (2010). Host interactions of probiotic bacterial surface molecules: comparison with commensals and pathogens. *Nat. Rev. Microbiol.* 8 171–184. 10.1038/nrmicro2297 20157338

[B69] Lee G. H. Ryu C.-M. (2016). Spraying of leaf-colonizing *Bacillus amyloliquefaciens* protects pepper from Cucumber mosaic virus. *Plant Dis.* 100 2099–2105. 10.1094/PDIS-03-16-0314-RE 30682996

[B70] Leveau J. H. J. Gerards S. (2008). Discovery of a bacterial gene cluster for catabolism of the plant hormone indole 3-acetic acid. *FEMS Microbiol. Ecol.* 65 238–250. 10.1111/j.1574-6941.2008.00436.x 18205812

[B71] Leveau J. H. J. Lindow S. E. (2005). Utilization of the plant hormone indole-3-acetic acid for growth by *Pseudomonas putida* strain 1290. *Appl. Environ. Microbiol.* 71 2365–2371. 10.1128/AEM.71.5.2365-2371.2005 15870323 PMC1087548

[B72] Li Z. Yao Q. Guo X. Crits-Christoph A. Mayes M. A. Iv W. J. H. (2019). Genome-resolved proteomic stable isotope probing of soil microbial communities using 13CO2 and 13C-Methanol. *Front. Microbiol.* 10:2706. 10.3389/fmicb.2019.02706 31866955 PMC6908837

[B73] Lin G. H. Chen H. P. Huang J. H. Liu T. T. Lin T. K. Wang S. J. (2012). Identification and characterization of an indigo-producing oxygenase involved in indole 3-acetic acid utilization by *Acinetobacter baumannii*. *Antonie van Leeuwenhoek* 101 881–890. 10.1007/s10482-012-9704-4 22311185

[B74] Lindow S. E. (1987). Competitive exclusion of epiphytic bacteria by Ice-*Pseudomonas syringae* mutants. *Appl. Environ. Microbiol.* 53 2520–2527. 10.1128/aem.53.10.2520-2527.1987 16347468 PMC204139

[B75] Luo L. Zhang Z. Wang P. Han Y. Jin D. Su P. (2019). Variations in phyllosphere microbial community along with the development of angular leaf-spot of cucumber. *AMB Express* 9:76. 10.1186/s13568-019-0800-y 31134393 PMC6536563

[B76] Madhaiyan M. Suresh Reddy B. V. Anandham R. Senthilkumar M. Poonguzhali S. Sundaram S. P. (2006). Plant growth-promoting Methylobacterium induces defense responses in groundnut (*Arachis hypogaea* L.) compared with rot pathogens. *Curr. Microbiol.* 53 270–276. 10.1007/s00284-005-0452-9 16941245

[B77] Maignien L. DeForce E. A. Chafee M. E. Murat Eren A. Simmons S. L. (2014). Ecological succession and stochastic variation in the assembly of Arabidopsis thaliana phyllosphere communities. *mBio* 5:e00682-13. 10.1128/mBio.00682-13 24449749 PMC3903271

[B78] Massart S. Margarita M. M. Jijakli M. H. (2015). Biological control in the microbiome era: challenges and opportunities. *Biol. Control* 89 98–108. 10.1016/j.biocontrol.2015.06.003

[B79] McClerklin S. A. Lee S. G. Harper C. P. Nwumeh R. Jez J. M. Kunkel B. N. (2018). Indole-3-acetaldehyde dehydrogenase-dependent auxin synthesis contributes to virulence of *Pseudomonas syringae* strain DC3000. *PLoS Pathog.* 14:e1006811. 10.1371/journal.ppat.1006811 29293681 PMC5766252

[B80] Mcdonald I. R. Murrell J. C. (1997). The methanol dehydrogenase structural gene mxaf and its use as a functional gene probe for methanotrophs and methylotrophs. *Appl. Environ. Microbiol.* 63 3218–3224. 10.1128/aem.63.8.3218-3224.1997 9251208 PMC168619

[B81] Mercier J. Lindow S. E. (2000). Role of leaf surface sugars in colonization of plants by bacterial epiphytes. *Appl. Environ. Microbiol.* 66 369–374. 10.1128/AEM.66.1.369-374.2000 10618250 PMC91832

[B82] Michavila G. Adler C. De Gregorio P. R. Lami M. J. Caram Di Santo M. C. Zenoff A. M. (2017). *Pseudomonas* protegens CS1 from the lemon phyllosphere as a candidate for citrus canker biocontrol agent. *Plant Biol.* 19 608–617. 10.1111/plb.12556 28194866

[B83] Miller E. R. Kearns P. J. Niccum B. A. Schwartz J. O. Ornstein A. Wolfe B. E. (2019). Establishment limitation constrains the abundance of lactic acid bacteria in the Napa cabbage phyllosphere. *Appl. Environ. Microbiol.* 85:AEM.00269-19. 10.1128/AEM.00269-19 31003989 PMC6581170

[B84] Morella N. M. Zhang X. Koskella B. (2019). Tomato seed-associated bacteria confer protection of seedlings against foliar disease caused by *Pseudomonas syringae*. *Phytobiomes J.* 3 177–190. 10.1094/PBIOMES-01-19-0007-R

[B85] Morohoshi T. Someya N. Ikeda T. (2009). Novel n-acylhomoserine lactone-degrading bacteria isolated from the leaf surface of solarium tuberosum and their quorum-quenching properties. *Biosci. Biotechnol. Biochem.* 73 2124–2127. 10.1271/bbb.90283 19734660

[B86] Nascimento F. X. Glick B. R. Rossi M. J. (2019). Isolation and characterization of novel soil-and plant-associated bacteria with multiple phytohormone-degrading activities using a targeted methodology. *Access Microbiol.* 1:e000053, 10.1099/acmi.0.000053 PMC7481731 32974544

[B87] Nascimento F. X. Rossi M. J. Glick B. R. (2018). Ethylene and 1-aminocyclopropane-1-carboxylate (ACC) in plant–bacterial interactions. *Front. Plant Sci.* 9:114. 10.3389/fpls.2018.00114 29520283 PMC5827301

[B88] Newman K. L. Chatterjee S. Ho K. A. Lindow S. E. (2008). Virulence of plant pathogenic bacteria attenuated by degradation of fatty acid cell-to-cell signaling factors. *Mol. Plant-Microbe Interact.* 21 326–334. 10.1094/MPMI-21-3-0326 18257682

[B89] Nielsen C. J. Ferrin D. M. Stanghellini M. E. (2006). Efficacy of biosurfactants in the management of *Phytophthora capsici* on pepper in recirculating hydroponic systems. *Can. J. Plant Pathol.* 28 450–460. 10.1080/07060660609507319

[B90] Nishimoto R. (2019). Global trends in the crop protection industry. *J. Pestic. Sci.* 44 141–147. 10.1584/jpestics.D19-101 31530972 PMC6718354

[B91] On A. Wong F. Ko Q. Tweddell R. J. Antoun H. Avis T. J. (2015). Antifungal effects of compost tea microorganisms on tomato pathogens. *Biol. Control* 80 63–69. 10.1016/J.BIOCONTROL.2014.09.017

[B92] Ona O. Van Impe J. Prinsen E. Vanderleyden J. (2005). Growth and indole-3-acetic acid biosynthesis of *Azospirillum brasilense* Sp245 is environmentally controlled. *FEMS Microbiol. Lett.* 246 125–132. 10.1016/j.femsle.2005.03.048 15869971

[B93] Ongena M. Jacques P. (2008). Bacillus lipopeptides: versatile weapons for plant disease biocontrol. *Trends Microbiol.* 16 115–125. 10.1016/j.tim.2007.12.009 18289856

[B94] Ongena M. Jourdan E. Adam A. Paquot M. Brans A. Joris B. (2007). Surfactin and fengycin lipopeptides of *Bacillus subtilis* as elicitors of induced systemic resistance in plants. *Environ. Microbiol.* 9 1084–1090. 10.1111/j.1462-2920.2006.01202.x 17359279

[B95] Park C.-J. Kim K.-J. Shin R. Park J. M. Shin Y.-C. Paek K.-H. (2004). Pathogenesis-related protein 10 isolated from hot pepper functions as a ribonuclease in an antiviral pathway. *Plant J.* 37 186–198. 10.1046/j.1365-313X.2003.01951 14690503

[B96] Pfeilmeier S. Caly D. L. Malone J. G. (2016). Bacterial pathogenesis of plants: future challenges from a microbial perspective: challenges in bacterial molecular plant pathology. *Mol. Plant Pathol.* 17 1298–1313. 10.1111/mpp.12427 27170435 PMC6638335

[B97] Pieterse C. M. J. Zamioudis C. Berendsen R. L. Weller D. M. Van Wees S. C. M. Bakker P. A. H. M. (2014). Induced systemic resistance by beneficial microbes. *Annu. Rev. Phytopathol.* 52 347–375. 10.1146/annurev-phyto-082712-102340 24906124

[B98] Pontonio E. Di Cagno R. Tarraf W. Filannino P. De Mastro G. Gobbetti M. (2018). Dynamic and assembly of epiphyte and endophyte lactic acid bacteria during the life cycle of *Origanum vulgare* L. *Front. Microbiol.* 9:1372. 10.3389/fmicb.2018.01372 29997592 PMC6029521

[B99] Radhika V. Ueda N. Tsuboi Y. Kojima M. Kikuchi J. Kudo T. (2015). Methylated Cytokinins from the Phytopathogen *Rhodococcus fascians* Mimic Plant Hormone Activity 1[OPEN]. *Plant Physiol.* 169 1118–1126. 10.1104/pp.15.00787 26251309 PMC4587462

[B100] Roitsch T. Ehneß R. (2000). Regulation of source/sink relations by cytokinins. *Plant Growth Regulat.* 32 359–367.

[B101] Romero F. M. Marina M. Pieckenstain F. L. (2016). Novel components of leaf bacterial communities of field-grown tomato plants and their potential for plant growth promotion and biocontrol of tomato diseases. *Res. Microbiol.* 167 222–233. 10.1016/j.resmic.2015.11.001 26654914

[B102] Ruiz J. A. Bernar E. M. Jung K. (2015). Production of siderophores increases resistance to fusaric acid in *Pseudomonas* protegens Pf-5. *PLoS One* 10:e0117040. 10.1371/journal.pone.0117040 25569682 PMC4287623

[B103] Ryffel F. Helfrich E. J. N. Kiefer P. Peyriga L. Portais J. C. Piel J. (2016). Metabolic footprint of epiphytic bacteria on Arabidopsis thaliana leaves. *ISME J.* 10 632–643. 10.1038/ismej.2015.141 26305156 PMC4817690

[B104] Saghafi D. Asgari Lajayer B. Ghorbanpour M. (2020). “Engineering bacterial ACC deaminase for improving plant productivity under stressful conditions,” in *Molecular Aspects of Plant Beneficial Microbes in Agriculture*, eds Sharma V. Salwan R. Tawfeeq Al-ani L. K. (Amsterdam: Elsevier), 259–277. 10.1016/b978-0-12-818469-1.00022-5

[B105] Saijo Y. Loo E. P. Yasuda S. (2018). Pattern recognition receptors and signaling in plant–microbe interactions. *Plant J.* 93 592–613. 10.1111/tpj.13808 29266555

[B106] Salvatierra-Martinez R. Arancibia W. Araya M. Aguilera S. Olalde V. Bravo J. (2018). Colonization ability as an indicator of enhanced biocontrol capacity—An example using two *Bacillus amyloliquefaciens* strains and *Botrytis cinerea* infection of tomatoes. *J. Phytopathol.* 166 601–612. 10.1111/jph.12718

[B107] Sanchez L. Courteaux B. Hubert J. Kauffmann S. Renault J. H. Clément C. (2012). Rhamnolipids elicit defense responses and induce disease resistance against biotrophic, hemibiotrophic, and necrotrophic pathogens that require different signaling pathways in Arabidopsis and highlight a central role for salicylic acid. *Plant Physiol.* 160 1630–1641. 10.1104/pp.112.201913 22968829 PMC3490604

[B108] Santos Kron A. Zengerer V. Bieri M. Dreyfuss V. Sostizzo T. Schmid M. (2020). *Pseudomonas orientalis* F9 Pyoverdine, Safracin, and Phenazine Mutants Remain Effective Antagonists against *Erwinia amylovora* in apple flowers. *Appl. Environ. Microbiol.* 86:e02620-19. 10.1128/AEM.02620-19 32033956 PMC7117935

[B109] Savary S. Willocquet L. Pethybridge S. J. Esker P. McRoberts N. Nelson A. (2019). The global burden of pathogens and pests on major food crops. *Nat. Ecol. Evol.* 3 430–439. 10.1038/s41559-018-0793-y 30718852

[B110] Schauer S. Kutschera U. (2011). A novel growth-promoting microbe, *Methylobacterium funariae* sp. nov., isolated from the leaf surface of a common moss. *Plant Signal. Behav.* 6 510–515. 10.4161/psb.6.4.14335 21673511 PMC3142378

[B111] Schauer S. Kutschera U. (2013). Methylobacteria isolated from bryophytes and the 2-fold description of the same microbial species. *Plant Signal. Behav.* 8:e23091. 10.4161/psb.23091 23299423 PMC3657004

[B112] Schenk S. T. Schikora A. (2015). AHL-Priming functions via oxylipin and salicylic acid. *Front. Plant Sci.* 5:784. 10.3389/fpls.2014.00784 25642235 PMC4294120

[B113] Schisler D. A. Khan N. I. Boehm M. J. Slininger P. J. (2002). Greenhouse and field evaluation of biological control of Fusarium head blight on durum wheat. *Plant Dis.* 86 1350–1356. 10.1094/PDIS.2002.86.12.1350 30818440

[B114] Sieper T. Forczek S. Matucha M. Krämer P. Hartmann A. Schröder P. (2014). N-acyl-homoserine lactone uptake and systemic transport in barley rest upon active parts of the plant. *New Phytol.* 201 545–555. 10.1111/nph.12519 24102510

[B115] Simionato A. S. Navarro M. O. P. de Jesus M. L. A. Barazetti A. R. da Silva C. S. Simões G. C. (2017). The effect of phenazine-1-carboxylic acid on mycelial growth of Botrytis cinerea produced by *Pseudomonas aeruginosa* LV strain. *Front. Microbiol.* 8:1102. 10.3389/fmicb.2017.01102 28659907 PMC5469906

[B116] Singh B. K. Trivedi P. (2017). Microbiome and the future for food and nutrient security. *Microb. Biotechnol.* 10 50–53. 10.1111/1751-7915.12592 28074557 PMC5270726

[B117] Smets W. Wuyts K. Oerlemans E. Wuyts S. Denys S. Samson R. (2016). Impact of urban land use on the bacterial phyllosphere of ivy (*Hedera* sp.). *Atmos. Environ.* 147 376–383. 10.1016/j.atmosenv.2016.10.017

[B118] Smits T. H. M. Duffy B. Blom J. Ishimaru C. A. Stockwell V. O. (2019). Pantocin A, a peptide-derived antibiotic involved in biological control by plant-associated *Pantoea* species. *Arch. Microbiol.* 201 713–722. 10.1007/s00203-019-01647-7 30868174

[B119] Stes E. Prinsen E. Holsters M. Vereecke D. (2012). Plant-derived auxin plays an accessory role in symptom development upon *Rhodococcus fascians* infection. *Plant J.* 70 513–527. 10.1111/j.1365-313X.2011.04890.x 22181713

[B120] Stockwell V. O. Johnson K. B. Sugar D. Loper J. E. (2002). Antibiosis contributes to biological control of fire blight by *Pantoea agglomerans* strain Eh252 in orchards. *Phytopathology* 92 1202–1209. 10.1094/PHYTO.2002.92.11.1202 18944246

[B121] Strano C. P. Bella P. Licciardello G. Caruso A. Catara V. (2017). Role of secondary metabolites in the biocontrol activity of *Pseudomonas corrugata* and *Pseudomonas mediterranea*. *Eur. J. Plant Pathol.* 149 103–115. 10.1007/s10658-017-1169-x

[B122] Stringlis I. A. Zamioudis C. Berendsen R. L. Bakker P. A. H. M. Pieterse C. M. J. (2019). Type III secretion system of beneficial rhizobacteria *Pseudomonas simiae* WCS417 and *Pseudomonas defensor* WCS374. *Front. Microbiol.* 10:1631. 10.3389/fmicb.2019.01631 31379783 PMC6647874

[B123] Surico G. Iacobellis N. S. Sisto A. (1985). Studies on the role of indole-3-acetic acid and cytokinins in the formation of knots on olive and oleander plants by *Pseudomonas syringae* pv. savastanoi. *Physiol. Plant Pathol.* 26 309–320. 10.1016/0048-4059(85)90006-2

[B124] Trias R. Bañeras L. Badosa E. Montesinos E. (2008). Bioprotection of Golden Delicious apples and Iceberg lettuce against foodborne bacterial pathogens by lactic acid bacteria. *Int. J. Food Microbiol.* 123 50–60. 10.1016/j.ijfoodmicro.2007.11.065 18191266

[B125] Tsang D. L. Edmond C. Harrington J. L. Nühse T. S. (2011). Cell wall integrity controls root elongation via a general 1-aminocyclopropane-1-carboxylic acid-dependent, ethylene-independent pathway. *Plant Physiol.* 156 596–604. 10.1104/pp.111.175372 21508182 PMC3177261

[B126] Tyagi S. Mulla S. I. Lee K. J. Chae J. C. Shukla P. (2018). VOCs-mediated hormonal signaling and crosstalk with plant growth promoting microbes. *Crit. Rev. Biotechnol.* 38 1277–1296. 10.1080/07388551.2018.1472551 29862848

[B127] Vanderhoef L. N. Dute R. R. (1981). Auxin-regulated wall loosening and sustained growth in elongation. *Plant Physiol.* 67 146–149. 10.1104/pp.67.1.146 16661616 PMC425639

[B128] Vanderstraeten L. van Der Straeten D. (2017). Accumulation and transport of 1-aminocyclopropane-1-carboxylic acid (ACC) in plants: current status, considerations for future research and agronomic applications. *Front. Plant Sci.* 8:38. 10.3389/fpls.2017.00038 28174583 PMC5258695

[B129] Veliz E. A. Martínez-Hidalgo P. Hirsch A. M. (2017). Chitinase-producing bacteria and their role in biocontrol. *AIMS Microbiol.* 3 689–705. 10.3934/microbiol.2017.3.689 31294182 PMC6604996

[B130] Vogel C. Bodenhausen N. Gruissem W. Vorholt J. A. (2016). The Arabidopsis leaf transcriptome reveals distinct but also overlapping responses to colonization by phyllosphere commensals and pathogen infection with impact on plant health. *New Phytol.* 212 192–207. 10.1111/nph.14036 27306148

[B131] Völksch B. May R. (2001). Biological control of *Pseudomonas syringae* pv. glycinea by epiphytic bacteria under field conditions. *Microb. Ecol.* 41 132–139. 10.1007/s002480000078 12032618

[B132] Vorholt J. A. (2012). Microbial life in the phyllosphere. *Nat. Rev. Microbiol.* 10 828–840. 10.1038/nrmicro2910 23154261

[B133] Walterson A. M. Smith D. D. N. Stavrinides J. (2014). Identification of a Pantoea biosynthetic cluster that directs the synthesis of an antimicrobial natural product. *PLoS One* 9:e96208. 10.1371/journal.pone.0096208 24796857 PMC4010436

[B134] Walterson A. M. Stavrinides J. (2015). Pantoea: insights into a highly versatile and diverse genus within the *Enterobacteriaceae*. *FEMS Microbiol. Rev.* 39 968–984. 10.1093/femsre/fuv027 26109597

[B135] Wang J. Liu J. Chen H. Yao J. (2007). Characterization of Fusarium graminearum inhibitory lipopeptide from *Bacillus subtilis* IB. *Appl. Microbiol. Biotechnol.* 76 889–894. 10.1007/s00253-007-1054-1 17611753

[B136] Wei F. Hu X. Xu X. (2016). Dispersal of *Bacillus subtilis* and its effect on strawberry phyllosphere microbiota under open field and protection conditions. *Sci. Rep.* 6:22611. 10.1038/srep22611 26936109 PMC4776175

[B137] Wensing A. Braun S. D. Büttner P. Expert D. Völksch B. Ullrich M. S. (2010). Impact of siderophore production by *Pseudomonas syringae* pv. syringae 22d/93 on epiphytic fitness and biocontrol Activity against *Pseudomonas syringae* pv. glycinea 1a/96. *Appl. Environ. Microbiol.* 76 2704–2711. 10.1128/AEM.02979-09 20208028 PMC2863448

[B138] Wilson M. Lindow S. E. (1994). Coexistence among epiphytic bacterial populations mediated through nutritional resource partitioning. *Appl. Environ. Microbiol.* 60 4468–4477. 10.1128/aem.60.12.4468-4477.1994 16349462 PMC202007

[B139] Wu L. Wu H. Chen L. Yu X. Borriss R. Gao X. (2015). Difficidin and bacilysin from *Bacillus amyloliquefaciens* FZB42 have antibacterial activity against Xanthomonas oryzae rice pathogens. *Sci. Rep.* 5 1–9. 10.1038/srep12975 26268540 PMC4534799

[B140] Xin X. F. Nomura K. Aung K. Velásquez A. C. Yao J. Boutrot F. (2016). Bacteria establish an aqueous living space in plants crucial for virulence. *Nature* 539 524–529. 10.1038/nature20166 27882964 PMC5135018

[B141] Yang T. Wei Z. Friman V. P. Xu Y. Shen Q. Kowalchuk G. A. (2017). Resource availability modulates biodiversity-invasion relationships by altering competitive interactions. *Environ. Microbiol.* 19 2984–2991. 10.1111/1462-2920.13708 28229529

[B142] Yasmin S. Hafeez F. Y. Mirza M. S. Rasul M. Arshad H. M. I. Zubair M. (2017). Biocontrol of Bacterial Leaf Blight of rice and profiling of secondary metabolites produced by rhizospheric *Pseudomonas aeruginosa* BRp3. *Front. Microbiol.* 8:1895. 10.3389/fmicb.2017.01895 29018437 PMC5622989

[B143] Zeriouh H. de Vicente A. Pérez-García A. Romero D. (2014). Surfactin triggers biofilm formation of *Bacillus subtilis* in melon phylloplane and contributes to the biocontrol activity. *Environ. Microbiol.* 16 2196–2211. 10.1111/1462-2920.12271 24308294

[B144] Zeriouh H. Romero D. García-Gutiérrez L. Cazorla F. M. De Vicente A. Pérez-García A. (2011). The Iturin-like lipopeptides are essential components in the biological control arsenal of bacillus subtilis against bacterial diseases of cucurbits. *Mol. Plant-Microbe Interact.* 24 1540–1552. 10.1094/MPMI-06-11-0162 22066902

